# A database of geopositioned Middle East Respiratory Syndrome Coronavirus occurrences

**DOI:** 10.1038/s41597-019-0330-0

**Published:** 2019-12-13

**Authors:** Rebecca E. Ramshaw, Ian D. Letourneau, Amy Y. Hong, Julia Hon, Julia D. Morgan, Joshua C. P. Osborne, Shreya Shirude, Maria D. Van Kerkhove, Simon I. Hay, David M. Pigott

**Affiliations:** 1 Institute for Health Metrics and Evaluation, University of Washington, 2301 5th Ave., Suite 600, Seattle, WA United States; 2 Bloomberg School of Public Health, Johns Hopkins University, 615N Wolfe St, Baltimore, MD 21205 United States; 3 Department of Infectious Hazards Management, Health Emergencies Programme, World Health Organization, Avenue Appia 20, 1211 Geneva, Switzerland; 4 Department of Health Metrics Sciences, School of Medicine, University of Washington, 2301 5th Ave., Suite 600, Seattle, WA United States

**Keywords:** Research data, Diseases

## Abstract

As a World Health Organization Research and Development Blueprint priority pathogen, there is a need to better understand the geographic distribution of Middle East Respiratory Syndrome Coronavirus (MERS-CoV) and its potential to infect mammals and humans. This database documents cases of MERS-CoV globally, with specific attention paid to zoonotic transmission. An initial literature search was conducted in PubMed, Web of Science, and Scopus; after screening articles according to the inclusion/exclusion criteria, a total of 208 sources were selected for extraction and geo-positioning. Each MERS-CoV occurrence was assigned one of the following classifications based upon published contextual information: index, unspecified, secondary, mammal, environmental, or imported. In total, this database is comprised of 861 unique geo-positioned MERS-CoV occurrences. The purpose of this article is to share a collated MERS-CoV database and extraction protocol that can be utilized in future mapping efforts for both MERS-CoV and other infectious diseases. More broadly, it may also provide useful data for the development of targeted MERS-CoV surveillance, which would prove invaluable in preventing future zoonotic spillover.

## Abstract (summary)

**Table:** 

Measurement(s)	Middle East Respiratory Syndrome • geographic location
Technology Type(s)	digital curation
Factor Type(s)	geographic distribution of Middle East Respiratory Syndrome Coronavirus (MERS-CoV) • year
Sample Characteristic - Organism	Middle East respiratory syndrome-related coronavirus
Sample Characteristic - Location	Earth (planet)

Machine-accessible metadata file describing the reported data: 10.6084/m9.figshare.11108801

## Background & Summary

Middle East Respiratory Syndrome Coronavirus (MERS-CoV) emerged as a global health concern in 2012 when the first human case was documented in Saudi Arabia^1^. Now listed as one of the WHO Research and Development Blueprint priority pathogens, cases have been reported in 27 countries across four continents^2^. Imported cases into non-endemic countries such as France, Great Britain, the United States, and South Korea have caused secondary cases^3–5^, thus highlighting the potential for MERS-CoV to spread far beyond the countries where index cases originate. Reports in animals suggest that viral circulation could be far more widespread than suggested by human cases alone^6–8^.

To help prevent future incidence of MERS-CoV, public health officials can focus on mitigating zoonotic transfer; however, in order to do this effectively, additional research is needed to determine where spillover could occur between mammals and humans. Previous literature reviews have looked at healthcare-associated outbreaks^9^, importation events resulting in secondary cases^10,11^, occurrences among dromedary camels^12,13^, or to summarize current knowledge and knowledge gaps of MERS-CoV^14,15^. This database seeks fill gaps in literature and build upon existing notification data by enhancing the geographic resolution of MERS-CoV data and providing occurrences of both mammal and environmental detections in addition to human cases. This information can help inform epidemiological models and targeted disease surveillance, both of which play important roles in strengthening global health security. Knowledge of the geographic extent of disease transmission allows stakeholders to develop appropriate emergency response and preparedness activities (https://www.jeealliance.org/global-health-security-and-ihr-implementation/joint-external-evaluation-jee/), inform policy for livestock trade and quarantine, determine appropriate demand for future vaccines (http://cepi.net/mission) and decide where to deliver them. Additionally, targeted disease surveillance will provide healthcare workers with updated lists of at-risk countries. Patients with a history of travel to affected regions could then be rapidly isolated and treated, thus reducing risk of nosocomial transmission.

This database is comprised of 861 unique geo-positioned MERS-CoV occurrences extracted from reports published between October 2012 and February 2018. It systematically captures unique occurrences of MERS-CoV globally by geo-tagging published reports of MERS-CoV cases and detections. Data collection, database creation, and geo-tagging methods are described below. Instructions on how to access the database are provided as well, with the aim to contribute to future epidemiological analysis. All data is available from the Global Health Data Exchange^16^ and Figshare^17^.

## Methods

The methods and protocols summarized below have been adapted from previously published literature extraction processes^18–22^, and provide additional context surrounding our systematic data collection from published reports of MERS-CoV.

### Data collection

We identified published reports of MERS-CoV by searching PubMed, Web of Science, and Scopus with the following terms: “Middle Eastern Respiratory Syndrome”, “Middle East Respiratory Syndrome”, “MERSCoV”, and “MERS”. The initial search was for all articles published about MERS-CoV prior to April 30, 2017, and was subsequently updated to February 22, 2018. These searches were conducted through the University of Washington Libraries’ institutional database subscriptions. We searched the Web of Science Web of Science Core Collection (the subscribed edition includes Science Citation Index Expanded, 1900-present; Social Sciences Citation Index, 1975-present; Arts & Humanities Citation Index, 1975-present; Emerging Sources Citation Index, 2015-present). We searched the standard Scopus database and the standard, freely available PubMed database; these products have a single version that is consistent across institutional subscriptions or access points.

**Fig. 1:**
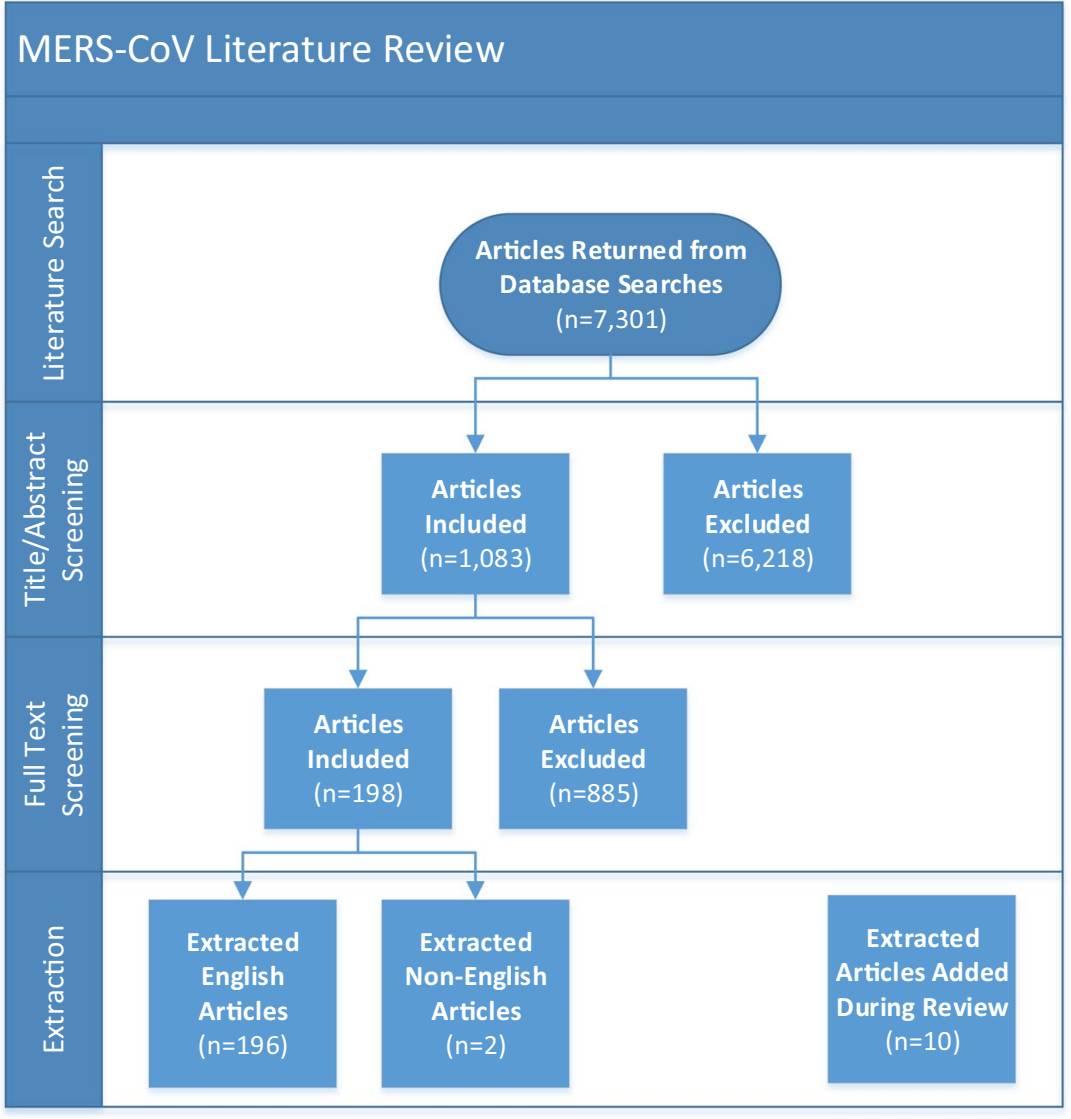
Middle East Respiratory Syndrome Coronavirus (MERS-CoV) literature extraction flowchart. Process of data source selection from initial literature search to extraction.

In total, this search returned 7,301 related abstracts, which were collated into a database before a title-abstract screening was manually conducted (Fig. 1. Flowchart). Articles were removed if they did not contain an occurrence of MERS-CoV; for example, vaccine development research or coronavirus proteomic analyses. Non-English articles were flagged for further review and brought into the full text screening stage. The accompanying supplementary file highlight the title and abstract screening process and the inclusion and exclusion criteria.

Full text review was conducted on 1,083 sources. To meet the inclusion criteria, articles must have contained both of the following items: 1) a detection of MERS-CoV from humans, animals, or environmental sources, and 2) MERS-CoV occurrences tagged with spatial information. Additionally, extractors attempted to prospectively manually remove articles containing duplicate occurrences that were already extracted in the dataset. Extractors only prospectively manually removed articles if it was clear the articles contained data we were confident had already been extracted and had high-quality data. We excluded 885 sources based on full text review. In addition, we reviewed citations and retroactively added relevant articles to our database if they were not already included. We retroactively added and subsequently marked ten articles for extraction using this process. In total, we extracted 208 peer-reviewed sources reporting detection of MERS-CoV that included geographic and relevant epidemiological metadata.

### Geo-positioning of data

Google Maps or ArcGIS^23^ was used to manually extract location information at the highest resolution available from individual articles. We evaluated spatial information as either points or polygons. The geography was defined as a point if the location of transmission was reported to have occurred within a 5 × 5 km area. Point data are represented by a specific latitude and longitude. A point references an area smaller than 5 × 5 km in order to be compatible with the typical 5 × 5 km resolution of satellite imagery used for global analyses.

The geography was defined as a polygon if the location of transmission was less clear, but known to have occurred in a general area (e.g. a province), or the location of transmission occurred within an area greater than 5 × 5 km (e.g. a large city). We used contextual information to determine location in instances where the author’s spelling of a location differed from Google Maps or ArcGIS. Maps provided by authors were digitized using ArcGIS.

We used three different types of polygons: known administrative boundaries, buffers, and custom polygons. Relevant administrative units were sourced from the Global Administrative Unit Layers curated by the Food and Agricultural Organization of the UN^24^ for known administrative boundaries of governorates, districts, or regions, and paired with the occurrence record. Buffers were created to encompass areas in cities and regions without corresponding administrative units. To ensure that buffers encompassed the entirety of the area of interest, Google Maps was used to determine the required radius. In areas with unspecified boundaries (e.g. Table Mountain National Park and the border region between Saudi Arabia and UAE) ArcGIS was used to generate custom polygons, which were assigned a unique code within a defined shapefile for ease of re-identification.

## Data Records

This database is publicly available online^16,17^. Each of the 861 rows represents a unique occurrence of MERS-CoV. Rows containing an index, unspecified, or imported case represent a single case of MERS-CoV. Rows containing mammal and secondary cases may represent more than one case but are still unique geospatial occurrences. Table 1 shows an overview of the content available in the publicly available dataset. In addition, online-only Table 1 lists occurrences by geography, origin, 405 shape type, and publication and online-only Table 2 provides citations of the data.

1. **nid**: A unique identifier assigned to each publication that was extracted
2. **title:** Title of the publication
3. **author**: Article’s author(s).
4. **doi:** Article’s DOI.
5. **abstract**: Article’s abstract, if available.
6. **source_title**: Journal in which the article was published.
7. **year**: Article’s publication year.
8. **source**: Database where article was found.
9. **pmid_if_applicable**: PMID if the article is from PubMed.
10. **full_text_link_if_included**: Link to the full text, if available.
11. **file_id**: Reference to pdf in format FirstAuthor_Year (e.g. Smith_2017).
12. **occ_id**: Unique identifier assigned to each occurrence of MERS-CoV. A single pdf may represent more than one occurrence. Each row will have its own occ_id, starting at 1 and numbered consecutively to 883.
13. **organism_type**: What type of organism tested positive for MERS-CoV (human, mammal, or environmental).
14. **organism_specific**: Specifies the exact organism that tested positive for MERS-CoV. Names are made consistent with Wilson and Reeder (2005) Mammal Species of the World^25^.
15. **pathogen**: Name the pathogen identified (e.g. MERS-CoV, Bat Coronaviruses, and other MERS-CoV-like pathogens).
16. **pathogen_note**: Miscellaneous notes regarding pathogen.
17. **patient_type**: index, unspecified, NA, secondary, import, or absent.

- index: Any human infection of MERS-CoV resulting after direct contact with an animal and no reported contact with a confirmed MERS-CoV case or healthcare setting.
- unspecified: Cases that lacked sufficient epidemiological evidence to classify them as any other status (e.g. serosurvey studies).
- NA: Non-applicable field; case was not a patient (e.g. mammal)
- secondary: Defined as any cases resulting from contact with known human infections. Cases reported after the index case can be assumed to be secondary cases unless accompanied by specific details of likely independent exposure to an animal reservoir.
- import: Cases that were brought into a non-endemic country after transmission occurred elsewhere.
- absent: Suspected case(s) ultimately confirmed negative for MERS-CoV.

18. **transmission_route**: zoonotic, direct, unspecified, or animal-to-animal.

- zoonotic: Transmission occurred from an animal to a human.
- direct: Only relevant for human-to-human transmission.
- unspecified: Lacked sufficient epidemiological evidence to classify a human case as zoonotic or direct.
- animal-to-animal: Transmission occurred from an animal to another animal.

19. **clinical**: Describes whether the MERS-CoV occurrence demonstrated clinical signs of infection. Denoted by yes, no, or unknown.

- yes: Clinical signs of infection were present/reported. Clinical signs among humans may range from mild (e.g. fever, cough) to severe (e.g. pneumonia, kidney failure). Clinical signs among camels include nasal discharge.
- no: Clinical signs of infection were not present/reported.
- unknown: Subject(s) may or may not have been demonstrating clinical signs of infection. For example, some authors did not explicitly mention symptoms, but individuals reportedly sought medical care. Another example being when a diagnostic serosurvey was conducted during an ongoing outbreak. The term “unknown” was used when articles lacked sufficient evidence for extractors to definitively label as “yes” or “no”.

20. **diagnostic**: Describes the class of diagnostic method that was used. PCR, serology, or reported.
21. **diagnostic_note**: More detailed information related to the specific test used (e.g. rk39, IgG, or IgM serology).
22. **serosurvey**: Describes the context if serological testing was used.

- diagnostic: testing of symptomatic patients.
- exploratory: historic exposure determined among healthy asymptomatic individuals.

23. **country**: ISO3 code for country in which the case occurred.
24. **origin**: Open-ended field to provide more details on the specific in-country location of MERS-CoV case.
25. **problem_geography**: This field was utilized if the MERS-CoV case was reported in a location that could cause uncertainty when determining exact geographic occurrence (e.g. hospital, abattoir).
26. **lat**: Latitude measured in decimal degrees.
27. **long**: Longitude measured in decimal degrees.
28. **latlong_source**: The source from which latitude and longitude were derived.
29. **loc_confidence**: States the level of confidence that researchers had when assigning a geographic location to the MERS-CoV case (good or bad). An answer of ‘good’ meant the article stated clearly that the case occurred in a specific geographic location and no assumptions were required on part of the researcher. An answer of ‘bad’ meant the article did not clearly state the specific geographic location of the MERS-CoV case, but the researcher was able to infer the location of occurrence. The field SITE_NOTES was utilized to detail the logic behind researchers’ decisions when inference was required.
30. **shape_type**: The geographic shape type assigned to the MERS-CoV occurrence (point or polygon).
31. **poly_type**: If the MERS-CoV occurrence was assigned a shape_type of polygon, was it admin (GAUL), custom, or buffer?
32. **buffer_radius**: If a MERS-CoV occurrence was assigned a buffer, what is the radius in km?
33. **gaul_year_or_custom_shapefile**: File path used to reach the necessary shape file in ArcGIS. Users of this dataset can find custom shapefiles created for this dataset at: https://cloud.ihme.washington.edu/index.php/s/DGoyKYqnbjG54F2/download
34. **poly_id**: A standardized and unique identifier assigned to each GAUL shapefile.
35. **poly_field**: Which type of polygon was used to geo-position the occurrence? (e.g. if admin1 polygon was used, enter ADM1_CODE)
36. **site_notes**: Miscellaneous notes regarding the site of occurrence.
37. **month_start**: Month that the occurrence(s) began. If the article provided a specific month of illness onset, the month was assigned a number from 1–12 (1 = January, 2 = February, etc.). If the article did not provide a specific month of illness onset, then researchers assigned a value of ‘NA’.
38. **month_end**: Month that the occurrence(s) ended, defined as the date a patient tested negative for MERS-CoV. If the article provided a specific month for recovery, the month was assigned a number from 1–12 (1 = January, 2 = February, etc.). If the article did not provide a specific month of symptom onset, then researchers assigned a value of ‘NA’.
39. **year_start**: Year that the occurrence(s) began. If the year of illness onset was not provided in the article, the IHME standard was used:
  (year_start = publication year – 3).
40. **year_end**: Year that the occurrence(s) ended. If the article did not provide a specific year for recovery, the IHME standard was used:
  (year_end = publication year – 1).
41. **year_accuracy**: If years were reported, this field was assigned a value of ‘0’. If assumptions were required, this field was assigned a value of ‘1’.

**Table 1:** Middle East Respiratory Syndrome Coronavirus (MERS-CoV) occurrences by patient type and geographic precision.

Data file	Points	Buffer	Custom	Admin2	Admin1	Admin0	Total
Index	34	99	1	0	93	7	234
Unspecified	86	50	1	4	35	27	203
Mammal	53	56	7	30	43	19	208
Import	11	2	0	2	10	9	34
Secondary	82	30	1	1	26	8	148
Absent	3	8	0	0	7	3	21
Environmental	0	1	0	0	0	2	3
MERS-CoV-like	1	1	7	0	0	1	10

**Fig. 2:**
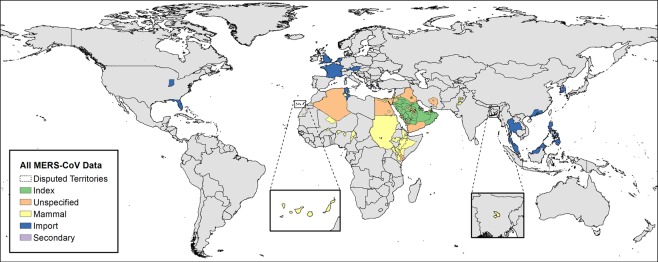
Geographic distribution of published detection of Middle East Respiratory Syndrome Coronavirus (MERS-CoV). Occurrences are layered from top to bottom in the following order: Index (green), Unspecified (orange), Mammal (yellow), Import (blue), Secondary (purple). Points were plotted using their assigned latitudes and longitudes, and shape files were created for polygons. Buffers were also plotted using assigned latitudes and longitudes, after which each buffer’s custom radius was drawn. Higher resolution geographies (points, buffers, governorates) were plotted on top of lower resolution geographies (countries, regions).

**Fig. 3:**
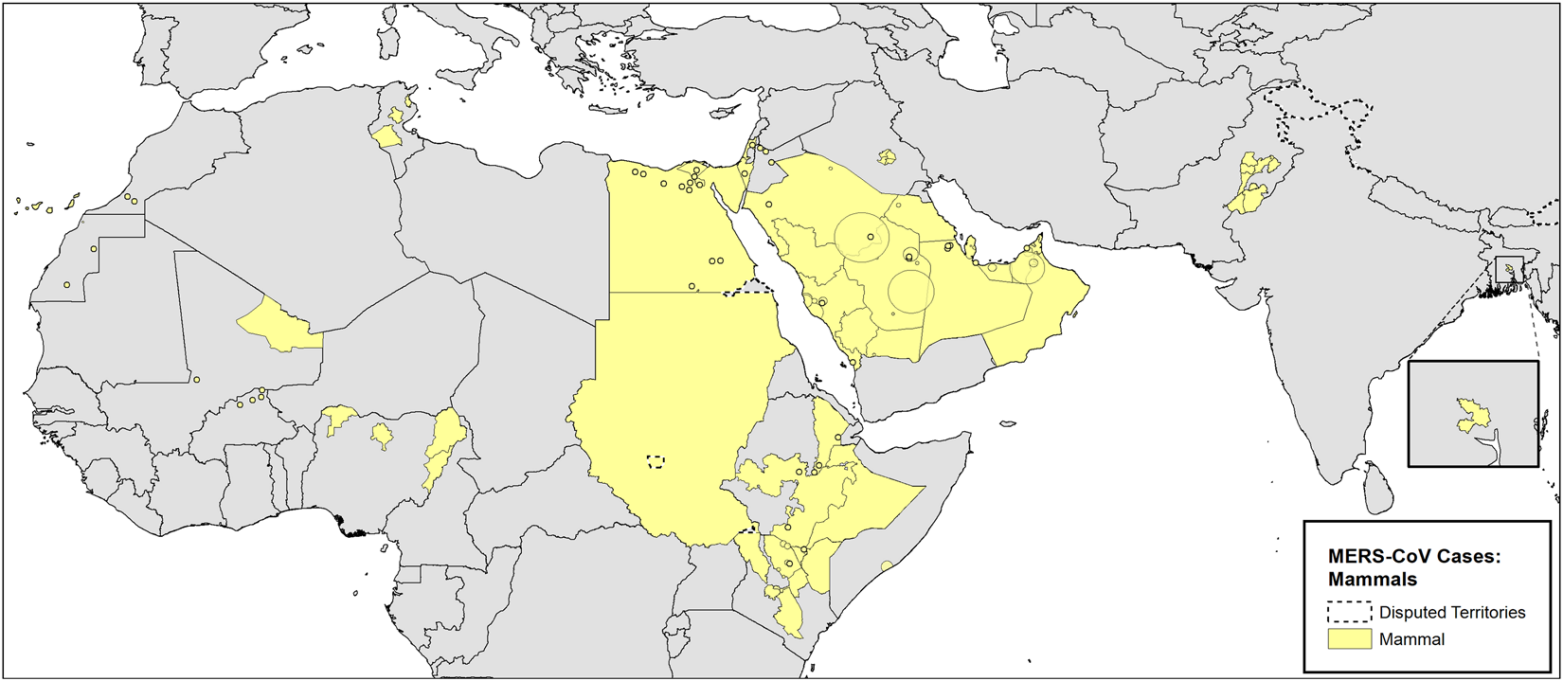
Geographic distribution of detections of Middle East Respiratory Syndrome Coronavirus (MERS-CoV) in mammals. Mammal populations testing positive for MERS-CoV primarily consisted of camels but also included a sheep, hamadryas baboon, Egyptian tomb bat, and an alpaca. Points were plotted using their assigned latitudes and longitudes, and shape files were created for polygons. Buffers were also plotted using assigned latitudes and longitudes, after which each buffer’s custom radius was drawn. Higher resolution geographies (points, buffers, governorates) were plotted on top of lower resolution geographies (countries, regions).

**Fig. 4:**
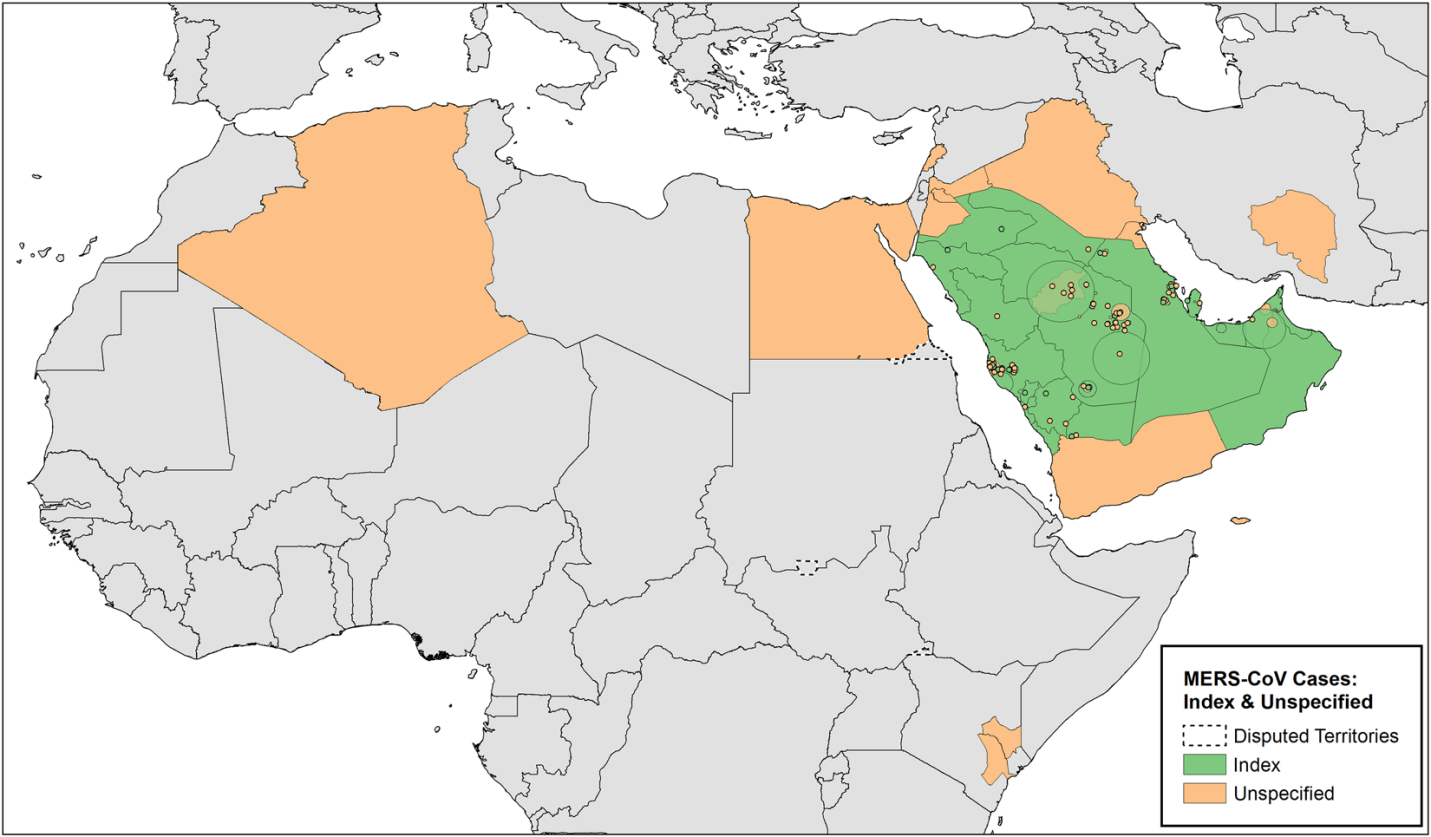
Geographic distribution of detections of Middle East Respiratory Syndrome Coronavirus (MERS-CoV) among cases tagged as Index or unspecified. Occurrences tagged as Index are coloured green, those tagged as unspecified are coloured orange. Points were plotted using their assigned latitudes and longitudes, and shape files were created for polygons. Buffers were also plotted using assigned latitudes and longitudes, after which each buffer’s custom radius was drawn. Higher resolution geographies (points, buffers, governorates) were plotted on top of lower resolution geographies (countries, regions).

Figures 2–4 show the geographic distribution of the MERS-CoV occurrence database, with distinctions made by epidemiological and geographic metadata.

## Technical Validation

All data extracted from the original search (October 2012 to April 30, 2017) was reviewed independently by a second individual to check for accuracy. Challenging extractions from the updated search (May 1, 2017 to February 22, 2018) were selected for group review during bi-weekly team meetings. Upon extraction completion, all data were checked to ensure they fell on land and within the correct country.

While the protocol implemented above was designed to reduce the amount of subjective decisions made by extractors, total elimination was not possible. Wherever a subjective decision had to be made, the extractor utilized the various notes fields in order to document the logic behind decisions. These decisions were subsequently reviewed by other extractors.

## Usage Notes

The techniques described here can be applied to collect and curate datasets for other infectious diseases, as has been previously demonstrated with dengue^20^ and leishmaniasis^18^. Additionally, since these data were collected independently through published reports of MERS-CoV occurrence, they may be used to build upon existing notification data^26,27^. Our ability to capture occurrences in this dataset is contingent on the data contained within published literature. Therefore, this dataset does not represent a total count of all cases. Instead, this dataset’s value lies within its geo-precision. Data were extracted with a focus on obtaining the highest resolution possible. These data may be merged with other datasets, such as WHO^26^ or OIE^27^ surveillance records, and are intended to complement, not replace, these resources. Together, published reports and notification data can provide a more comprehensive snapshot of current disease extent and at-risk locations.

An important consideration, whether using the literature data alone, or in combination with other databases, is the potential for duplication. Various pieces of metadata can be used to evaluate where potential duplicates could lie, such as common date fields (month_start, month_end, year_start, year_end) or consistent geographic details (lat, long, poly_id, shape_type) or shared epidemiological tags (patient_type). Researchers may wish to consider further steps, such as fuzzy matching of geographic data (e.g. matching a point with an overlapping buffer) or temporal data (e.g. matching a precise month with an overlapping month interval). We acknowledge this duplicate-removal process will not catch all matching records, but it will likely catch several. We recommend this approach because it will allow researchers to remove several duplicates without erroneously deleting any two occurrences that are truly unique (i.e. not duplicates). Essentially, we recommend a sensitive approach above a more specific approach, as the latter simply risks culling too many records that aren’t actually duplicates.

When merging with other databases, consistency in metadata tagging is essential. For the WHO Disease Outbreak News data feed^26,27^ for instance, nomenclature for case definitions is slightly different, with WHO definitions of “Community Acquired” and “Not Reported” comparable to “Index” and “Unspecified” respectively. In addition, it is important to recognize what information is beyond the scope of these additional databases. Again, when comparing to the WHO dataset, it is important to recognize that serologically positive cases do not meet the case definition used in the WHO database. These adjustments need to be identified on a dataset-to-dataset basis.

This database can be combined with other covariates (e.g. satellite imagery) to produce environmental suitability models of MERS-CoV infection risk and potential spillover on both global and regional scales as achieved with other exemplar datasets^28–31^. This information can be useful in resource allocation aimed at improving disease surveillance and contribute towards a better understanding of the factors facilitating continued emergence of index cases.

The addition of sampling techniques and prevalence data may improve this dataset. Researchers were ultimately unable to add these data due to inconsistencies in the way literature reported sampling techniques and prevalence date by geography. An attempt to extract these data using the current approach would have led to sporadic inclusion of this information and would not have been comprehensive for the entire dataset. Moving forward, we recommend authors report sampling technique and prevalence data at the highest resolution geography possible, as seen in Miguel *et al*.^32^. We encourage continued presentation of paired epidemiological and geographic metadata that would allow for more detailed analysis in the future.

This database may also be utilized in clinical settings to provide an evidence-base for diagnoses when used in conjunction with patient travel histories. Additionally, it can be used to identify geographies for surveillance, particularly areas where MERS-CoV has been documented in animals but not humans (e.g. Ethiopia and Nigeria). Identifying locations for surveillance will, in turn, inform global health security. While models will increase the resolution at which these questions can be addressed, datasets such as this provide an initial baseline.

A major limitation of this database is the potential for sampling bias, which stems from higher frequency of disease reporting within countries where there exists strong healthcare infrastructure and reporting systems. This database does not attempt to account for such biases, which must be addressed in subsequent modelling activities where such biases are of consequence. Similarly, another limitation is potential duplicate documentation of singular occurrences. This can happen when the same occurrence is assigned different geographies (e.g. point, polygon) in multiple publications. Even though extractors made efforts to prospectively manually identify duplicate occurrences, this was challenging because the process relied upon papers providing sufficient details for extractors to determine a duplicate occurrence (e.g. geography, patient demographics, dates of occurrence, diagnostic methods, etc.). However, the majority of papers did not report such details for each occurrence. In those instances, it was impossible for extractors to discern whether occurrences may have been duplicates from a previous artic le. Even case studies inconsistently reported patient details and demographic information. These are some examples of challenges faced by extractors when we attempted to identify duplicates. Without sufficient contextual clues, extractors lacked evidence to determine duplicity and thus likely extracted some unique occurrences more than once. Despite efforts to remove duplicate occurrences from the database, it is possible that some remain.

Geographic uncertainty is similarly problematic for analyses such as this. In some cases, polygons, as opposed to points, are utilised as a geographic frame of reference, reflecting the uncertainty in geotagging in the articles themselves. For some occurrences, there is a strong assumption that the geography listed corresponds to the site of infection. While the use of 5 km × 5 km as the minimum geographical unit allows for some leeway in this precision, it is possible that even with the point data (often corresponding to household clusters) these may not map directly with true infection sites. This must be considered in any subsequent geospatial analysis.

Finally, this database represents a time-bounded survey of the literature. While all efforts were made to be comprehensive within this period, articles, and therefore data, will continue to be published. Efforts to streamline ongoing collection processes are still to be fully realized^33^. Regardless, we hope that this dataset provides a solid baseline for further iteration.

### Supplementary information

Supplementary Table 1

## Online-only Tables

**Online-only Table 1:** MERS_CoV occurrences by geography, origin, 405 shape type, and publication.

Geography	(N)	Origin, Shape Type	Publication
Algeria	1	Human (unspecified), polygon	Leitmeyer, 2014^34^
Austria	1	Human (import), polygon	Kwok-ming *et al*., 2015^35^
Bahrain	1	Human (import), point	Seddiq_2017^36^
Bangladesh	1	Human (absent), polygon	Muraduzzaman *et al*., 2018^37^
	1	Camel, polygon	Islam *et al*., 2018^38^
Burkina Faso	4	Camel, point	Miguel *et al*., 2017^32^
Canary Islands	1	Camel, polygon	Reusken *et al*., 2013^39^
	1	Camel, polygon	Gutierrez *et al*., 2015^8^
	1	Human (absent), point	Rubio *et al*., 2018^40^
China	1	Human (import), polygon	Guan *et al*., 2015^41^
	1	Human (import), polygon	Lu *et al*., 2015^42^
	1	Human (import), polygon	Wu *et al*., 2015^43^
	1	Bat (MERS-like), polygon	Yang *et al*., 2014^44^
	1	Human (absent), polygon	Liu *et al*., 2017^45^
	1	Human (absent), polygon	Ma *et al*., 2017^46^
	1	Human (import), polygon	Xie *et al*., 2017^47^
	1	Human (import), polygon	Chen *et al*., 2017^48^
	1	Human (import), point	Ling *et al*., 2015^49^
	1	Human (absent), polygon	Liu *et al*., 2017^45^
Egypt	1	Camel, polygon	Kandeil *et al*., 2016^50^
	1	Camel, polygon	Ali *et al*., 2017^51^
	1	Sheep, polygon	
	1	Camel, polygon	Mueller *et al*., 2014^52^
	2	Camel, polygon	Chu *et al*., 2014^53^
	1	Camel, polygon	Perera *et al*., 2013^54^
	12	Camel, point	Ali *et al*., 2017^55^
	1	Human (unspecified), polygon	Al-Tawfiq & Memish, 2014^56^
Ethiopia	3	Camel, polygon	Reusken *et al*., 2014^57^
	5	Camel, point	Miguel *et al*., 2017^32^
France	1	Human (import), point	Guery *et al*., 2013^3^
	1	Human (secondary), point	
	1	Human (import), polygon	Mailles *et al*., 2013^58^
	1	Human (secondary), polygon	
Germany	1	Human (import), point	Drosten *et al*., 2013^59^
Great Britain	1	Human (import), polygon	Thomas *et al*., 2014^4^
	1	Human (secondary), polygon	
Greece	1	Human (import), point	Tsiodras *et al*., 2014^60^
	1	Human (import), point	Kossyvakis *et al*., 2015^61^
Iran	1	Human (unspecified), polygon	Yavarian *et al*., 2015^62^
	1	Human (secondary), polygon	
	1	Human (secondary), point	Yousefi *et al*., 2017^63^
	1	Human (secondary), polygon	Moniri *et al*., 2015^64^
	1	Human (absent), point	Yavarian *et al*., 2017^65^
	1	Human (absent), polygon	
Iraq	6	Camel, polygon	Al Salihi & Alrodhan, 2017^66^
	5	Human (unspecified), polygon	
Israel	1	Camel, polygon	David *et al*., 2018^67^
	1	Camel, point	
	1	Alpaca, point	
	1	Llama, point	
	2	Camel, polygon	Harcourt *et al*., 2018^68^
Italy	5	Bat (MERS-like), polygon	Lelli *et al*., 2013^69^
	1	Human (import), polygon	Puzelli *et al*., 2013^70^
	1	Human (secondary), polygon	
Jordan	1	Human (unspecified), polygon	Wickramage *et al*., 2013^71^
	2	Camel, point	van Doremalen *et al*., 2017^72^
	1	Human (unspecified), polygon	Puzelli *et al*., 2013^70^
	1	Human (secondary), polygon	Shalhoub *et al*., 2016^73^
	1	Camel, point	Reusken *et al*., 2013^74^
	1	Human (secondary), polygon	Payne *et al*., 2014^75^
Kenya	11	Camel, polygon	Corman *et al*., 2014^76^
	2	Human (unspecified), polygon	Liljander *et al*., 2016^77^
	2	Camel, point	Munyua *et al*., 2017^78^
	6	Camel, polygon	
	1	Camel, polygon	Deem *et al*., 2015^79^
Kuwait	4	Human (unspecified), polygon	Aly *et al*., 2017^80^
Lebanon	1	Human (unspecified), polygon	Aly *et al*., 2017^80^
	1	Human (unspecified), polygon	Sharif-Yakan & Kanj, 2014^81^
Malaysia	1	Human (import), polygon	Devi *et al*., 2014^82^
Mali	1	Camel, point	Falzarano *et al*., 2017^83^
	1	Camel, polygon	
Morocco	4	Camel, point	Miguel *et al*., 2017^32^
	1	Camel, polygon	
Netherlands	1	Human (import), polygon	Kraaij-Dirkzwager *et al*., 2014^84^
Nigeria	4	Camel, polygon	Reusken *et al*., 2014^57^
	1	Camel, polygon	Chu *et al*., 2015^85^
	1	Camel, polygon	So *et al*., 2018^86^
Oman	1	Human (index), polygon	Al Hammadi *et al*., 2015^87^
	1	Camel, polygon	
	1	Camel, polygon	Nowotny & Kolodziejek, 2014^88^
	1	Camel, polygon	Reusken *et al*., 2013^39^
	1	Human (index), polygon	Jahan & Al Maqbali, 2015^89^
	1	Human (index), polygon	Paden *et al*., 2017^90^
	1	Human (unspecified), polygon	Plipat *et al*., 2017^91^
Pakistan	15	Camel, polygon	Saqib *et al*., 2017^92^
Philippines	1	Human (import), polygon	Racelis *et al*., 2015^93^
Qatar	1	Human (unspecified), polygon	Wickramage *et al*., 2013^71^
	1	Human (unspecified), point	Varughese *et al*., 2015^94^
	2	Camel, polygon	Farag *et al*., 2015^95^
	1	Human (unspecified), polygon	Kwok-ming *et al*., 2015^35^
	1	Camel, polygon	Raj *et al*., 2014^96^
	1	Alpaca, polygon	Reusken *et al*., 2016^97^
	1	Camel, polygon	
	1	Camel, polygon	Reusken *et al*., 2014^98^
	2	Human (index), polygon	Haagmans *et al*., 2014^7^
	1	Camel, polygon	
	11	Human (index), polygon	Reusken *et al*., 2015^99^
	8	Human (index), point	
	9	Human (index), point	Sikkema *et al*., 2017^100^
Saudi Arabia	1	Human (unspecified), polygon	Wickramage *et al*., 2013^71^
	1	Human (index), polygon	Oboho *et al*., 2015^101^
	2	Human (secondary), polygon	
	1	Human (secondary), point	Tsiodras *et al*., 2014^60^
	1	Human (secondary), polygon	Al-Gethamy *et al*., 2015^102^
	1	Human (secondary), polygon	Garbati *et al*., 2016^103^
	1	Human (secondary), polygon	Omrani *et al*., 2013^104^
	1	Human (secondary), point	Khalid *et al*., 2016^105^
	1	Human (secondary), point	Das *et al*., 2015^106^
	1	Human (unspecified), point	Alhogbani, 2016^107^
	1	Human (index), polygon	El Bushra *et al*., 2016^108^
	1	Camel, polygon	
	1	Human (secondary), polygon	
	2	Human (secondary), point	Alshukairi *et al*., 2016^109^
	1	Human (unspecified), polygon	Memish, 2013^110^
	1	Human (secondary), polygon	
	1	Human (unspecified), point	Al-Hameed *et al*., 2016^111^
	1	Human (secondary), point	
	1	Human (secondary), polygon	Kapoor *et al*., 2014^112^
	1	Human (unspecified), point	Saad *et al*., 2014^113^
	2	Human (secondary), point	
	2	Human (unspecified), point	Arabi *et al*., 2014^114^
	2	Human (secondary), point	
	12	Camel, polygon	Sabir *et al*., 2016^115^
	2	Human (index), polygon	Memish *et al*., 2014^116^
	2	Human (secondary), polygon	
	1	Human (unspecified), polygon	Racelis *et al*., 2015^93^
	1	Human (unspecified), point	Balkhy *et al*., 2016^117^
	1	Human (secondary), point	
	8	Human (unspecified), polygon	Noorwali *et al*., 2015^118^
	8	Human (secondary), polygon	
	1	Environmental, polygon	Azhar *et al*., 2014^119^
	1	Human (index), point	Assiri *et al*., 2013^120^
	8	Human (unspecified), point	
	2	Human (secondary), point	
	10	Human (unspecified), polygon	
	1	Human (index), polygon	Azhar *et al*., 2014^121^
	1	Camel, polygon	
	3	Human (index), polygon	Alhakeem *et al*., 2016^122^
	2	Human (unspecified), polygon	
	5	Human (secondary), polygon	
	1	Human (unspecified), polygon	Bialek *et al*., 2014^123^
	1	Human (unspecified), polygon	Assiri *et al*., 2013^124^
	2	Human (secondary), polygon	
	1	Human (index), point	Memish *et al*., 2014^125^
	1	Camel, polygon	
	1	Human (unspecified), point	Alenazi *et al*., 2017^126^
	2	Human (secondary), point	
	1	Human (secondary), point	Alserehi *et al*., 2016^127^
	1	Human (unspecified), polygon	Kossyvakis *et al*., 2015^61^
	1	Human (index), polygon	Devi *et al*., 2014^82^
	1	Camel, polygon	Hemida *et al*., 2015^128^
	10	Camel, polygon	Hemida *et al*., 2017^129^
	8	Human (unspecified), point	Cotten *et al*., 2013^130^
	3	Human (unspecified), point	Cotten *et al*., 2014^131^
	5	Human (unspecified), point	Drosten *et al*., 2015^132^
	1	Human (unspecified), point	Assiri *et al*., 2016(a)^133^
	1	Human (unspecified), polygon	
	1	Human (secondary), polygon	
	23	Human (unspecified), point	Assiri *et al*., 2016(b)^134^
	2	Camel, polygon	Hemida *et al*., 2014^135^
	1	Human (unspecified), point	AlGhamdi *et al*., 2015^136^
	1	Human (secondary), point	
	1	Human (import), point	Shalhoub *et al*., 2016^73^
	1	Human (secondary), point	
	9	Camel, point	Khalafalla *et al*., 2015^137^
	2	Camel, point	Hemida *et al*., 2013^138^
	1	Human (unspecified), polygon	Kraaij-Dirkzwager *et al*., 2014^84^
	1	Human (secondary), polygon	
	2	Human (unspecified), polygon	Memish *et al*., 2015^139^
	2	Human (secondary), polygon	
	1	Human (index), polygon	Sherbini *et al*., 2017^140^
	1	Human (unspecified), polygon	
	1	Human (secondary), polygon	
	1	Human (unspecified), point	Thabet *et al*., 2015^141^
	3	Human (unspecified), polygon	Assiri *et al*., 2016^142^
	6	Camel, point	Alagaili *et al*., 2014^143^
	1	Human (secondary), point	Arwady *et al*., 2016^144^
	1	Human (unspecified), point	Khalid *et al*., 2014^145^
	2	Human (secondary), point	
	1	Human (unspecified), polygon	Bayrakdar *et al*., 2015^146^
	1	Human (unspecified), point	Fagbo *et al*., 2015^147^
	2	Human (secondary), point	
	1	Human (index), point	Zaki *et al*., 2012^1^
	1	Human (unspecified), point	Balkhy *et al*., 2016^148^
	2	Human (secondary), point	
	1	Human (secondary), point	Hastings *et al*., 2016^149^
	2	Human (secondary), point	Alraddadi *et al*., 2016^150^
	2	Human (unspecified), point	Mohd *et al*., 2016^151^
	6	Human (unspecified), polygon	Müller *et al*., 2015^152^
	1	Human (unspecified), point	Almekhlafi *et al*., 2016^153^
	4	Human (unspecified), point	Motabi *et al*., 2016^154^
	13	Human (index), point	Alraddadi *et al*., 2016^155^
	2	Human (secondary), point	
	1	Human (secondary), point	Al-Hameed, 2017^156^
	1	Human (secondary), point	Shalhoub *et al*., 2015^157^
	10	Human (unspecified), polygon	Bin Saeed *et al*., 2017^158^
	1	Human (unspecified), point	Al-Dorzi *et al*., 2016^159^
	1	Human (secondary), point	
	1	Human (unspecified), polygon	Park *et al*., 2015^160^
	1	Human (secondary), polygon	Fanoy *et al*., 2014^161^
	1	Human (unspecified), point	Al Ghamdi *et al*., 2016^162^
	2	Human (secondary), point	
	2	Human (secondary), point	Alfaraj *et al*., 2018^163^
	1	Human (unspecified), point	Alsaad *et al*., 2018^164^
	1	Human (secondary), point	Al-Tawfiq & Hinedi, 2018^165^
	9	Camel, polygon	Kasem *et al*., 2018^166^
	1	Human (unspecified), polygon	Paden *et al*., 2017^90^
	1	Human (unspecified), point	Alhetheel *et al*., 2017^167^
	1	Human (secondary), point	
	167	Human (index), polygon	Kasem *et al*., 2017^168^
	12	Camel, polygon	
	10	Camel (absent), polygon	
	1	Human (unspecified), point	Al-Tawfiq *et al*., 2017^169^
	2	Human (unspecified), polygon	Lippold *et al*., 2017^170^
	4	Human (unspecified), point	Zhao *et al*., 2017^171^
	2	Human (secondary), point	Nazer, 2017^172^
	1	Human (secondary), polygon	
	1	Human (absent), polygon	Alrashid *et al*., 2017^173^
	1	Baboon (absent), polygon	Olarinmoye *et al*., 2017^174^
	1	Human (unspecified), point	Al-Tawfiq *et al*., 2017^175^
	1	Human (unspecified), point	Alfaraj *et al*., 2017^176^
	1	Human (secondary), point	
	23	Human (unspecified), polygon	El Bushra *et al*., 2017^177^
	5	Human (unspecified), point	Aleanizy *et al*., 2017^178^
	12	Human (unspecified), polygon	
	1	Human (index), polygon	Seddiq *et al*., 2017^36^
	1	Camel, polygon	Harrath & Abu Duhier, 2018^179^
	4	Camel, polygon	Hemida *et al*., 2014^180^
	13	Human (index), point	Alraddadi *et al*., 2016^181^
	1	Human (unspecified), polygon	Memish *et al*., 2014^182^
	1	Human (secondary), polygon	
Somalia	2	Camel, polygon	Müller *et al*., 2014^52^
South Africa	1	Bat (MERS-like), polygon	Ithete *et al*., 2013^183^
	1	Bat (MERS-like), polygon	Corman *et al*., 2014^184^
South Korea	17	Human (secondary), point	Ki, 2015^185^
	1	Human (import), point	Cha, *et al*., 2016^186^
	1	Human (import), polygon	The Korean Society of Infectious Diseases, and Korean Society for Healthcare-associated Infection Control and Prevention^187^
	1	Human (secondary), point	Kim *et al*., 2017^188^
	1	Human (secondary), point	Rhee *et al*., 2016^189^
	1	Human (secondary), polygon	Lu *et al*., 2015^42^
	1	Human (import), point	Park *et al*., 2016^5^
	1	Human (secondary), point	
	1	Human (secondary), point	Park *et al*., 2016^190^
	1	Human (secondary), point	Kim *et al*., 2015^191^
	1	Human (secondary), polygon	Choi *et al*., 2015^192^
	2	Environmental, polygon	Kim *et al*., 2016^193^
	1	Bat (MERS-like), polygon	Kim *et al*., 2016^194^
	1	Human (secondary), point	Nam *et al*., 2017^195^
	1	Human (secondary), polygon	
	1	Human (secondary), polygon	Wu *et al*., 2015^43^
	1	Human (secondary), point	Moon & Son, 2017^196^
	1	Human (secondary), polygon	Chang *et al*., 2015^197^
	1	Human (secondary), point	Cho *et al*., 2016^198^
	1	Human (import), polygon	Yang *et al*., 2015^199^
	1	Human (secondary), polygon	
	1	Human (secondary), point	Choi *et al*., 2016^200^
	1	Human (secondary), polygon	Park *et al*., 2016^201^
	1	Human (secondary), point	Kim *et al*., 2016^202^
	1	Human (secondary), point	Bae, 2015^203^
	1	Human (import), polygon	Park *et al*., 2015^160^
	1	Human (secondary), point	Kim *et al*., 2016^204^
	1	Human (secondary), point	Ko *et al*., 2018^205^
	1	Human (import), point	Xiao *et al*., 2018^206^
	1	Human (secondary), point	
	1	Human (absent), point	Lee *et al*., 2017^207^
	1	Human (secondary), point	Go *et al*., 2017^208^
	1	Human (secondary), point	Ko *et al*., 2018^209^
	1	Human (secondary), polygon	Xie *et al*., 2017^47^
	1	Human (import), polygon	Lee *et al*., 2017^210^
	3	Human (secondary), point	Choe *et al*., 2017^211^
	1	Human (secondary), point	Kim *et al*., 2017^212^
	3	Human (secondary), polygon	Jeong *et al*., 2017^213^
	1	Human (absent), polygon	
	2	Human (secondary), polygon	Park *et al*., 2017^214^
	1	Human (secondary), polygon	Ling *et al*., 2015^49^
Sudan	1	Camel, polygon	Ali *et al*., 2017^51^
	1	Camel, polygon	Müller *et al*., 2014^52^
Thailand	1	Human (import), point	Plipat *et al*., 2017^91^
	1	Human (unspecified), polygon	Wiboonchutikul *et al*., 2017^215^
Tunisia	1	Human (import), polygon	Abroug *et al*., 2014^216^
	1	Human (secondary), polygon	
	3	Camel, polygon	Reusken *et al*., 2014^57^
Turkey	1	Human (import), polygon	Bayrakdar *et al*., 2015^146^
Uganda	1	Bat (MERS-like), point	Anthony *et al*., 2017^217^
United Arab Emirates	1	Human (unspecified), polygon	Wickramage *et al*., 2013^71^
	1	Camel, polygon	Wernery *et al*., 2015a^218^
	1	Camel, polygon	Wernery *et al*., 2015b^219^
	1	Camel, point	Meyer *et al*., 2014^220^
	2	Camel, polygon	
	1	Human (index), polygon	Al Hammadi *et al*., 2015^87^
	1	Human (unspecified), polygon	Guery *et al*., 2013^3^
	1	Human (unspecified), point	Drosten *et al*., 2013^59^
	1	Human (unspecified), polygon	Ng *et al*., 2016^221^
	2	Human (index), polygon	Al Muhairi *et al*., 2016^222^
	2	Camel, polygon	
	1	Human (unspecified), polygon	Mailles *et al*., 2013^58^
	1	Camel, polygon	Alexandersen et a., 2014^223^
	1	Human (index), point	Malik *et al*., 2016^224^
	1	Human (secondary), polygon	
	6	Camel, polygon	Lau *et al*., 2016^225^
	1	Camel, polygon	Yusof *et al*., 2015^226^
	2	Camel, polygon	Meyer *et al*., 2016^227^
	2	Human (secondary), polygon	Hunter *et al*., 2016^228^
	2	Human (unspecified), polygon	Al Hosani *et al*., 2016^229^
	2	Human (secondary), polygon	
	3	Human (index), polygon	Paden *et al*., 2017^90^
	1	Human (unspecified), polygon	
	4	Human (secondary), polygon	
	1	Human (import), polygon	
	3	Camel, polygon	
	1	Camel, polygon	Yusof *et al*., 2017^230^
	1	Camel, polygon	Li *et al*., 2017^231^
	1	Human (unspecified), point	Habib *et al*., 2015^232^
	1	Camel, point	Wernery *et al*., 2014^233^
USA	1	Human (import), polygon	Kapoor *et al*., 2014^112^
	1	Human (import), polygon	Bialek *et al*., 2014^123^
	2	Human (import), polygon	Lippold *et al*., 2017^170^
Yemen	1	Human (unspecified), polygon	Buliva *et al*., 2017^234^

**Online-only Table 2:** data citation table.

NID	Geography	Dataset URL	Citation	Reference Number
364643	Tunisia		Abroug F, Slim A, Ouanes-Besbes L, Hadj Kacem MA, Dachraoui F, Ouanes I, Lu X, Tao Y, Paden C, Caidi H, Miao C, Al-Hajri MM, Zorraga M, Ghaouar W, BenSalah A, Gerber SI, World Health Organization Global Outbreak Alert and Response Network Middle East Respiratory Syndrome Coronavirus International Investigation Team. Family cluster of Middle East respiratory syndrome coronavirus infections, Tunisia, 2013. Emerg Infect Dis. 2014; 20(9): 1527-30.	^216^
365053	Saudi Arabia		Al Ghamdi M, Alghamdi KM, Ghandoora Y, Alzahrani A, Salah F, Alsulami A, Bawayan MF, Vaidya D, Perl TM, Sood G. Treatment outcomes for patients with Middle Eastern Respiratory Syndrome Coronavirus (MERS CoV) infection at a coronavirus referral center in the Kingdom of Saudi Arabia. BMC Infect Dis. 2016; 16: 174.	^162^
364310	Oman		Al Hammadi ZM, Chu DK, Eltahir YM, Al Hosani F, Al Mulla M, Tarnini W, Hall AJ, Perera RA, Abdelkhalek MM, Peiris JS, Al Muhairi SS, Poon LL. Asymptomatic MERS-CoV Infection in Humans Possibly Linked to Infected Dromedaries Imported from Oman to United Arab Emirates, May 2015. Emerg Infect Dis. 2015; 21(12): 2197-200.	^87^
365056	United Arab Emirates		Al Hosani FI, Pringle K, Al Mulla M, Kim L, Pham H, Alami NN, Khudhair A, Hall AJ, Aden B, El Saleh F, Al Dhaheri W, Al Bandar Z, Bunga S, Abou Elkheir K, Tao Y, Hunter JC, Nguyen D, Turner A, Pradeep K, Sasse J, Weber S, Tong S, Whitaker BL, Haynes LM, Curns A, Gerber SI. Response to Emergence of Middle East Respiratory Syndrome Coronavirus, Abu Dhabi, United Arab Emirates, 2013–2014. Emerg Infect Dis. 2016; 22(7): 1162-8.	^229^
412592	Iraq		Al Salihi SF, Alrodhan MA. Phylogenetic Analysis of MERSCoV in Human and Camels in Iraq. *International Journal of Pharmaceutical Research Allied Sciences*. 2017; 6(1): 1209.	^66^
364922	Saudi Arabia		Alagaili AN, Briese T, Mishra N, Kapoor V, Sameroff SC, Burbelo PD, de Wit E, Munster VJ, Hensley LE, Zalmout IS, Kapoor A, Epstein JH, Karesh WB, Daszak P, Mohammed OB, Lipkin WI. Middle East respiratory syndrome coronavirus infection in dromedary camels in Saudi Arabia. MBio. 2014; 5(2): e00884-14.	^143^
365039	Saudi Arabia		Al-Dorzi HM, Aldawood AS, Khan R, Baharoon S, Alchin JD, Matroud AA, Al Johany SM, Balkhy HH, Arabi YM. The critical care response to a hospital outbreak of Middle East respiratory syndrome coronavirus (MERS-CoV) infection: an observational study. Ann Intensive Care. 2016; 6(1): 101.	^159^
365157	Saudi Arabia		Aleanizy FS, Mohmed N, Alqahtani FY, El Hadi Mohamed RA. Outbreak of Middle East respiratory syndrome coronavirus in Saudi Arabia: a retrospective study. BMC Infect Dis. 2017; 17(1): 23.	^178^
364789	Saudi Arabia		Alenazi TH, Al Arbash H, El-Saed A, Alshamrani MM, Baffoe-Bonnie H, Arabi YM, Al Johani SM, Hijazi R, Alothman A, Balkhy HH. Identified Transmission Dynamics of Middle East Respiratory Syndrome Coronavirus Infection During an Outbreak: Implications of an Overcrowded Emergency Department. Clin Infect Dis. 2017; 65(4): 675–679.	^126^
364905	United Arab Emirates		Alexandersen S, Kobinger GP, Soule G, Wernery U. Middle East respiratory syndrome coronavirus antibody reactors among camels in Dubai, United Arab Emirates, in 2005. Transbound Emerg Dis. 2014; 61(2): 105-8.	^223^
365058	Philippines		Alfaraj SH, Al-Tawfiq JA, Altuwaijri TA, Alanazi M, Alzahrani N, Memish ZA. Middle East respiratory syndrome coronavirus transmission among health care workers: Implication for infection control. Am J Infect Control. 2018; 46(2): 165–168.	^163^
365186	Saudi Arabia		Alfaraj SH, Al-Tawfiq JA, Altuwaijri TA, Memish ZA. Middle East Respiratory Syndrome Coronavirus and Pulmonary Tuberculosis Coinfection: Implications for Infection Control. Intervirology. 2017; 60(1–2): 53–5.	^176^
364275	Saudi Arabia		Al-Gethamy M, Corman VM, Hussain R, Al-Tawfiq JA, Drosten C, Memish ZA. A case of long-term excretion and subclinical infection with Middle East respiratory syndrome coronavirus in a healthcare worker. Clin Infect Dis. 2015; 60(6): 973-4.	^102^
364874	Saudi Arabia		AlGhamdi M, Mushtaq F, Awn N, Shalhoub S. MERS CoV infection in two renal transplant recipients: case report. Am J Transplant. 2015; 15(4): 1101-4.	^136^
364640	Saudi Arabia		Alhakeem RF, Midgley CM, Assiri AM, Alessa M, Al Hawaj H, Saeed AB, Almasri MM, Lu X, Abedi GR, Abdalla O, Mohammed M, Algarni HS, Al-Abdely HM, Alsharef AA, Nooh R, Erdman DD, Gerber SI, Watson JT. Exposures among MERS Case-Patients, Saudi Arabia, January-February 2016. Emerg Infect Dis. 2016; 22(11): 2020–2022.	^122^
364316	Saudi Arabia		Al-Hameed F, Wahla AS, Siddiqui S, Ghabashi A, Al-Shomrani M, Al-Thaqafi A, Tashkandi Y. Characteristics and Outcomes of Middle East Respiratory Syndrome Coronavirus Patients Admitted to an Intensive Care Unit in Jeddah, Saudi Arabia. J Intensive Care Med. 2016; 31(5): 344-8.	^111^
365027	Saudi Arabia		Al-Hameed FM. Spontaneous intracranial hemorrhage in a patient with Middle East respiratory syndrome corona virus. Saudi Med J. 2017; 38(2): 196–200.	^156^
365087	Saudi Arabia		Alhetheel A, Altalhi H, Albarrag A, Shakoor Z, Mohamed D, El-Hazmi M, Somily A, Barry M, Bakhrebah M, Nassar M. Assessing the Detection of Middle East Respiratory Syndrome Coronavirus IgG in Suspected and Proven Cases of Middle East Respiratory Syndrome Coronavirus Infection. Viral Immunol. 2017; 30(9): 649–653.	^167^
364296	Saudi Arabia		Alhogbani T. Acute myocarditis associated with novel Middle east respiratory syndrome coronavirus. Ann Saudi Med. 2016; 36(1): 78–80.	^107^
364349	Egypt		Ali M, El-Shesheny R, Kandeil A, Shehata M, Elsokary B, Gomaa M, Hassan N, El Sayed A, El-Taweel A, Sobhy H, Fasina FO, Dauphin G, El Masry I, Wolde AW, Daszak P, Miller M, VonDobschuetz S, Morzaria S, Lubroth J, Makonnen YJ. Cross-sectional surveillance of Middle East respiratory syndrome coronavirus (MERS-CoV) in dromedary camels and other mammals in Egypt, August 2015 to January 2016. Euro Surveill. 2017; 22(11).	^51^
365037	Egypt		Ali MA, Shehata MM, Gomaa MR, Kandeil A, El-Shesheny R, Kayed AS, El-Taweel AN, Atea M, Hassan N, Bagato O, Moatasim Y, Mahmoud SH, Kutkat O, Maatouq AM, Osman A, McKenzie PP, Webby RJ, Kayali G. Systematic, active surveillance for Middle East respiratory syndrome coronavirus in camels in Egypt. Emerg Microbes Infect. 2017; 6(1): e1.	^55^
364965	Saudi Arabia		Almekhlafi GA, Albarrak MM, Mandourah Y, Hassan S, Alwan A, Abudayah A, Altayyar S, Mustafa M, Aldaghestani T, Alghamedi A, Talag A, Malik MK, Omrani AS, Sakr Y. Presentation and outcome of Middle East respiratory syndrome in Saudi intensive care unit patients. Crit Care. 2016; 20(1): 123.	^153^
364954	Saudi Arabia		Alraddadi B, Bawareth N, Omar H, Alsalmi H, Alshukairi A, Qushmaq I, Feteih M, Qutob M, Wali G, Khalid I. Patient characteristics infected with Middle East respiratory syndrome coronavirus infection in a tertiary hospital. Ann Thorac Med. 2016; 11(2): 128-31.	^150^
365016	Saudi Arabia		Alraddadi BM, Al-Salmi HS, Jacobs-Slifka K, Slayton RB, Estivariz CF, Geller AI, Al-Turkistani HH, Al-Rehily SS, Alserehi HA, Wali GY, Alshukairi AN, Azhar EI, Haynes L, Swerdlow DL, Jernigan JA, Madani TA. Risk Factors for Middle East Respiratory Syndrome Coronavirus Infection among Healthcare Personnel. Emerg Infect Dis. 2016; 22(11): 1915–1920.	^155^
365018	Saudi Arabia		Alraddadi BM, Watson JT, Almarashi A, Abedi GR, Turkistani A, Sadran M, Housa A, Almazroa MA, Alraihan N, Banjar A, Albalawi E, Alhindi H, Choudhry AJ, Meiman JG, Paczkowski M, Curns A, Mounts A, Feikin DR, Marano N, Swerdlow DL, Gerber SI, Hajjeh R, Madani TA. Risk Factors for Primary Middle East Respiratory Syndrome Coronavirus Illness in Humans, Saudi Arabia, 2014. Emerg Infect Dis. 2016; 22(1): 49–55.	^181^
365118	Saudi Arabia		Alrashid M, Taleb AA, Hajeer A, Arabi Y. Prevalence of antibodies against the Middle East Respiratory Syndrome coronavirus, influenza A and B viruses among blood donors, Saudi Arabia. Ann Thorac Med. 2017; 12(3): 217–218.	^173^
365484	Saudi Arabia		Alsaad KO, Hajeer AH, Al Balwi M, Al Moaiqel M, Al Oudah N, Al Ajlan A, AlJohani S, Alsolamy S, Gmati GE, Balkhy H, Al-Jahdali HH, Baharoon SA, Arabi YM. Histopathology of Middle East respiratory syndrome coronovirus (MERS-CoV) infection - clinicopathological and ultrastructural study. Histopathol. 2018; 72(3): 516-24.	^164^
364791	Saudi Arabia		Alserehi H, Wali G, Alshukairi A, Alraddadi B. Impact of Middle East Respiratory Syndrome coronavirus (MERS-CoV) on pregnancy and perinatal outcome. BMC Infect Dis. 2016; 16: 105.	^127^
364307	Saudi Arabia		Alshukairi AN, Khalid I, Ahmed WA, Dada AM, Bayumi DT, Malic LS, Althawadi S, Ignacio K, Alsalmi HS, Al-Abdely HM, Wali GY, Qushmaq IA, Alraddadi BM, Perlman S. Antibody Response and Disease Severity in Healthcare Worker MERS Survivors. Emerg Infect Dis. 2016; 22(6).	^109^
365095	Saudi Arabia		Al-Tawfiq JA, Alfaraj SH, Altuwaijri TA, Memish ZA. A cohort-study of patients suspected for MERS-CoV in a referral hospital in Saudi Arabia. J Infect. 2017; 75(4): 378–379.	^169^
365133	Saudi Arabia		Al-Tawfiq JA, Hinedi K, Abbasi S, Babiker M, Sunji A, Eltigani M. Hematologic, hepatic, and renal function changes in hospitalized patients with Middle East respiratory syndrome coronavirus. Int J Lab Hematol. 2017; 39(3): 272–278.	^175^
365060	Saudi Arabia		Al-Tawfiq JA, Hinedi K. The calm before the storm: clinical observations of Middle East respiratory syndrome (MERS) patients. J Chemother. 2018; 30(3): 179–182.	^165^
365166	North Africa and Middle East		Al-Tawfiq JA, Memish ZA. Middle East respiratory syndrome coronavirus: epidemiology and disease control measures. Infect Drug Resist. 2014; 7: 281–7.	^56^
365160	Bahrain, Kuwait, Oman, Qatar, Saudi Arabia, United Arab Emirates		Aly M, Elrobh M, Alzayer M, Aljuhani S, Balkhy H. Occurrence of the Middle East Respiratory Syndrome Coronavirus (MERS-CoV) across the Gulf Corporation Council countries: Four years update. PLoS One. 2017; 12(10): e0183850.	^80^
364646	Uganda		Anthony SJ, Gilardi K, Menachery VD, Goldstein T, Ssebide B, Mbabazi R, Navarrete-Macias I, Liang E, Wells H, Hicks A, Petrosov A, Byarugaba DK, Debbink K, Dinnon KH, Scobey T, Randell SH, Yount BL, Cranfield M, Johnson CK, Baric RS, Lipkin WI, Mazet JA. Further Evidence for Bats as the Evolutionary Source of Middle East Respiratory Syndrome Coronavirus. MBio. 2017; 8(2).	^217^
364324	Saudi Arabia		Arabi YM, Arifi AA, Balkhy HH, Najm H, Aldawood AS, Ghabashi A, Hawa H, Alothman A, Khaldi A, Al Raiy B. Clinical course and outcomes of critically ill patients with Middle East respiratory syndrome coronavirus infection. Ann Intern Med. 2014; 160(6): 389-97.	^114^
364926	Saudi Arabia		Arwady MA, Alraddadi B, Basler C, Azhar EI, Abuelzein E, Sindy AI, Sadiq BM, Althaqafi AO, Shabouni O, Banjar A, Haynes LM, Gerber SI, Feikin DR, Madani TA. Middle East Respiratory Syndrome Coronavirus Transmission in Extended Family, Saudi Arabia, 2014. Emerg Infect Dis. 2016; 22(8): 1395-402.	^144^
364919	Saudi Arabia		Assiri A, Abedi GR, Al Masri M, Bin Saeed A, Gerber SI, Watson JT. Middle East Respiratory Syndrome Coronavirus Infection During Pregnancy: A Report of 5 Cases From Saudi Arabia. Clin Infect Dis. 2016; 63(7): 951-3.	^142^
364844	Saudi Arabia		Assiri A, Abedi GR, Bin Saeed AA, Abdalla MA, al-Masry M, Choudhry AJ, Lu X, Erdman DD, Tatti K, Binder AM, Rudd J, Tokars J, Miao C, Alarbash H, Nooh R, Pallansch M, Gerber SI, Watson JT. Multifacility Outbreak of Middle East Respiratory Syndrome in Taif, Saudi Arabia. Emerg Infect Dis. 2016; 22(1): 32–40.	^133^
364636	Saudi Arabia		Assiri A, Al-Tawfiq JA, Al-Rabeeah AA, Al-Rabiah FA, Al-Hajjar S, Al-Barrak A, Flemban H, Al-Nassir WN, Balkhy HH, Al-Hakeem RF, Makhdoom HQ, Zumla AI, Memish ZA. Epidemiological, demographic, and clinical characteristics of 47 cases of Middle East respiratory syndrome coronavirus disease from Saudi Arabia: a descriptive study. Lancet Infect Dis. 2013; 13(9): 752-61.	^120^
364714	Saudi Arabia		Assiri A, McGeer A, Perl TM, Price CS, Al Rabeeah AA, Cummings DA, Alabdullatif ZN, Assad M, Almulhim A, Makhdoom H, Madani H, Alhakeem R, Al-Tawfiq JA, Cotten M, Watson SJ, Kellam P, Zumla AI, Memish ZA, KSA MERS-CoV Investigation Team. Hospital outbreak of Middle East respiratory syndrome coronavirus. N Engl J Med. 2013; 369(5): 407-16.	^124^
364637	Saudi Arabia		Assiri AM, Midgley CM, Abedi GR, Bin Saeed A, Almasri MM, Lu X, Al-Abdely HM, Abdalla O, Mohammed M, Algarni HS, Alhakeem RF, Sakthivel SK, Nooh R, Alshayab Z, Alessa M, Srinivasamoorthy G, AlQahtani SY, Kheyami A, HajOmar WH, Banaser TM, Esmaeel A, Hall AJ, Curns AT, Tamin A, Alsharef AA, Erdman D, Watson JT, Gerber SI. Epidemiology of a Novel Recombinant Middle East Respiratory Syndrome Coronavirus in Humans in Saudi Arabia. J Infect Dis. 2016; 214(5): 712-21.	^134^
364639	Saudi Arabia		Azhar EI, El-Kafrawy SA, Farraj SA, Hassan AM, Al-Saeed MS, Hashem AM, Madani TA. Evidence for camel-to-human transmission of MERS coronavirus. N Engl J Med. 2014; 370(26): 2499-505.	^121^
364583	Saudi Arabia		Azhar EI, Hashem AM, El-Kafrawy SA, Sohrab SS, Aburizaiza AS, Farraj SA, Hassan AM, Al-Saeed MS, Jamjoom GA, Madani TA. Detection of the Middle East respiratory syndrome coronavirus genome in an air sample originating from a camel barn owned by an infected patient. MBio. 2014; 5(4): e01450-14.	^119^
365035	South Korea		Bae JM. Surveillance operation for the 141st confirmed case of Middle East Respiratory Syndrome coronavirus in response to the patient’s prior travel to Jeju Island. Epidemiol Health. 2015; 37: e2015035.	^203^
364943	Saudi Arabia		Balkhy HH, Alenazi TH, Alshamrani MM, Baffoe-Bonnie H, Al-Abdely HM, El-Saed A, Al Arbash HA, Al Mayahi ZK, Assiri AM, Bin Saeed A. Notes from the Field: Nosocomial Outbreak of Middle East Respiratory Syndrome in a Large Tertiary Care Hospital–Riyadh, Saudi Arabia, 2015. MMWR Morb Mortal Wkly Rep. 2016; 65(6): 163-4.	^148^
364351	Saudi Arabia		Balkhy HH, Alenazi TH, Alshamrani MM, Baffoe-Bonnie H, Arabi Y, Hijazi R, Al-Abdely HM, El-Saed A, Al Johani S, Assiri AM, Bin Saeed A. Description of a Hospital Outbreak of Middle East Respiratory Syndrome in a Large Tertiary Care Hospital in Saudi Arabia. Infect Control Hosp Epidemiol. 2016; 37(10): 1147-55.	^117^
364935	Turkey		Bayrakdar F, Altaş AB, Korukluoğlu G, Topal S. [Molecular diagnosis and phylogenetic analysis of the first MERS case in Turkey]. Mikrobiyol Bul. 2015; 49(3): 414-22.	^146^
365195	United States	https://www.cdc.gov/mmwr/preview/mmwrhtml/mm6319a4.htm	Bialek SR, Allen D, Alvarado-Ramy F, Arthur R, Balajee A, Bell D, Best S, Blackmore C, Breakwell L, Cannons A, Brown C, Cetron M, Chea N, Chommanard C, Cohen N, Conover C, Crespo A, Creviston J, Curns AT, Dahl R, Dearth S, DeMaria A, Echols F, Erdman DD, Feikin D, Frias M, Gerber SI, Gulati R, Hale C, Haynes LM, Heberlein-Larson L, Holton K, Ijaz K, Kapoor M, Kohl K, Kuhar DT, Kumar AM, Kundich M, Lippold S, Liu L, Lovchik JC, Madoff L, Martell S, Matthews S, Moore J, Murray LR, Onofrey S, Pallansch MA, Pesik N, Pham H, Pillai S, Pontones P, Pringle K, Pritchard S, Rasmussen S, Richards S, Sandoval M, Schneider E, Schuchat A, Sheedy K, Sherin K, Swerdlow DL, Tappero JW, Vernon MO, Watkins S, Watson J. First Confirmed Cases of Middle East Respiratory Syndrome Coronavirus (MERS-CoV) Infection in the United States, Updated Information on the Epidemiology of MERS-CoV Infection, and Guidance for the Public, Clinicians, and Public Health Authorities May 2014. MMWR Morb Mortal Wkly Rep. 2014; 63(19): 431-436.	^123^
365164	North Africa and Middle East		Buliva E, Elhakim M, Tran Minh NN, Elkholy A, Mala P, Abubakar A, Malik SMMR. Emerging and Reemerging Diseases in the World Health Organization (WHO) Eastern Mediterranean Region-Progress, Challenges, and WHO Initiatives. Front Public Health. 2017; 5: 276.	^234^
364277	South Korea		Cha RH, Yang SH, Moon KC, Joh JS, Lee JY, Shin HS, Kim DK, Kim YS. A Case Report of a Middle East Respiratory Syndrome Survivor with Kidney Biopsy Results. J Korean Med Sci. 2016; 31(4): 635-40.	^186^
364875	South Korea		Chang K, Ki M, Lee EG, Lee SY, Yoo B, Choi JH. MERS epidemiological investigation to detect potential mode of transmission in the 178th MERS confirmed case in Pyeongtaek, Korea. Epidemiol Health. 2015; 37: e2015036.	^197^
365129	China		Chen Z, Bao L, Chen C, Zou T, Xue Y, Li F, Lv Q, Gu S, Gao X, Cui S, Wang J, Qin C, Jin Q. Human Neutralizing Monoclonal Antibody Inhibition of Middle East Respiratory Syndrome Coronavirus Replication in the Common Marmoset. J Infect Dis. 2017; 215(12): 1807–1815.	^48^
364886	South Korea		Cho SY, Kang JM, Ha YE, Park GE, Lee JY, Ko JH, Lee JY, Kim JM, Kang CI, Jo IJ, Ryu JG, Choi JR, Kim S, Huh HJ, Ki CS, Kang ES, Peck KR, Dhong HJ, Song JH, Chung DR, Kim YJ. MERS-CoV outbreak following a single patient exposure in an emergency room in South Korea: an epidemiological outbreak study. Lancet. 2016; 388(10048): 994–1001.	^198^
365120	South Korea		Choe PG, Perera RAPM, Park WB, Song KH, Bang JH, Kim ES, Kim HB, Ko LWR, Park SW, Kim NJ, Lau EHY, Poon LLM, Peiris M, Oh MD. MERS-CoV Antibody Responses 1 Year after Symptom Onset, South Korea, 2015. Emerg Infect Dis. 2017; 23(7): 1079–1084.	^211^
364634	South Korea		Choi JH, Yoo B, Lee SY, Lee EG, Ki M, Lee W, Jung JR, Chang K. Epidemiological investigation of the 119th confirmed Middle East Respiratory Syndrome coronavirus case with an indefinite mode of transmission during the Pyeongtaek outbreak in Korea. Epidemiol Health. 2015.	^192^
364931	South Korea		Choi WJ, Lee KN, Kang EJ, Lee H. Middle East Respiratory Syndrome-Coronavirus Infection: A Case Report of Serial Computed Tomographic Findings in a Young Male Patient. Korean J Radiol. 2016; 17(1): 166-70.	^200^
364892	Nigeria		Chu DK, Oladipo JO, Perera RA, Kuranga SA, Chan SM, Poon LL, Peiris M. Middle East respiratory syndrome coronavirus (MERS-CoV) in dromedary camels in Nigeria, 2015. Euro Surveill. 2015; 20(49).	^85^
364872	Egypt		Chu DK, Poon LL, Gomaa MM, Shehata MM, Perera RA, Abu Zeid D, El Rifay AS, Siu LY, Guan Y, Webby RJ, Ali MA, Peiris M, Kayali G. MERS coronaviruses in dromedary camels, Egypt. Emerg Infect Dis. 2014; 20(6): 1049-53.	^53^
365020	South Africa		Corman VM, Ithete NL, Richards LR, Schoeman MC, Preiser W, Drosten C, Drexler JF. Rooting the phylogenetic tree of middle East respiratory syndrome coronavirus by characterization of a conspecific virus from an African bat. J Virol. 2014; 88(19): 11297-303.	^184^
364305	Kenya		Corman VM, Jores J, Meyer B, Younan M, Liljander A, Said MY, Gluecks I, Lattwein E, Bosch BJ, Drexler JF, Bornstein S, Drosten C, Müller MA. Antibodies against MERS coronavirus in dromedary camels, Kenya, 1992-2013. Emerg Infect Dis. 2014; 20(8): 1319-22.	^76^
364839	Saudi Arabia		Cotten M, Watson SJ, Kellam P, Al-Rabeeah AA, Makhdoom HQ, Assiri A, Al-Tawfiq JA, Alhakeem RF, Madani H, AlRabiah FA, Al Hajjar S, Al-nassir WN, Albarrak A, Flemban H, Balkhy HH, Alsubaie S, Palser AL, Gall A, Bashford-Rogers R, Rambaut A, Zumla AI, Memish ZA. Transmission and evolution of the Middle East respiratory syndrome coronavirus in Saudi Arabia: a descriptive genomic study. Lancet. 2013; 382(9909): 1993–2002.	^130^
364840	Saudi Arabia		Cotten M, Watson SJ, Zumla AI, Makhdoom HQ, Palser AL, Ong SH, Al Rabeeah AA, Alhakeem RF, Assiri A, Al-Tawfiq JA, Albarrak A, Barry M, Shibl A, Alrabiah FA, Hajjar S, Balkhy HH, Flemban H, Rambaut A, Kellam P, Memish ZA. Spread, circulation, and evolution of the Middle East respiratory syndrome coronavirus. MBio. 2014; 5(1).	^131^
364292	Saudi Arabia		Das KM, Lee EY, Al Jawder SE, Enani MA, Singh R, Skakni L, Al-Nakshabandi N, AlDossari K, Larsson SG. Acute Middle East Respiratory Syndrome Coronavirus: Temporal Lung Changes Observed on the Chest Radiographs of 55 Patients. AJR Am J Roentgenol. 2015; 205(3): W267-74.	^106^
394849	Israel		David D, Rotenberg D, Khinich E, Erster O, Bardenstein S, van Straten M, Okba NMA, Raj SV, Haagmans BL, Miculitzki M, Davidson I. Middle East respiratory syndrome coronavirus specific antibodies in naturally exposed Israeli llamas, alpacas and camels. One Health. 2018; 5: 65–68.	^67^
365025	Kenya		Deem SL, Fèvre EM, Kinnaird M, Browne AS, Muloi D, Godeke GJ, Koopmans M, Reusken CB. Serological Evidence of MERS-CoV Antibodies in Dromedary Camels (Camelus dromedaries) in Laikipia County, Kenya. PLoS One. 2015; 10(10): e0140125.	^79^
364842	Saudi Arabia		Drosten C, Muth D, Corman VM, Hussain R, Al Masri M, HajOmar W, Landt O, Assiri A, Eckerle I, Al Shangiti A, Al-Tawfiq JA, Albarrak A, Zumla A, Rambaut A, Memish ZA. An observational, laboratory-based study of outbreaks of middle East respiratory syndrome coronavirus in Jeddah and Riyadh, kingdom of Saudi Arabia, 2014. Clin Infect Dis. 2015; 60(3): 369-77.	^132^
365170	Germany		Drosten C, Seilmaier M, Corman VM, Hartmann W, Scheible G, Sack S, Guggemos W, Kallies R, Muth D, Junglen S, Müller MA, Haas W, Guberina H, Röhnisch T, Schmid-Wendtner M, Aldabbagh S, Dittmer U, Gold H, Graf P, Bonin F, Rambaut A, Wendtner C-M. Clinical features and virological analysis of a case of Middle East respiratory syndrome coronavirus infection. Lancet Infect Dis. 2013; 13(9): 745–51.	^59^
364299	Saudi Arabia		El Bushra HE, Abdalla MN, Al Arbash H, Alshayeb Z, Al-Ali S, Latif ZA, Al-Bahkit H, Abdalla O, Mohammed M, Al-Abdely H, Chahed M, Lohiniva AL, Saeed AB. An outbreak of Middle East Respiratory Syndrome (MERS) due to coronavirus in Al-Ahssa Region, Saudi Arabia, 2015. East Mediterr Health J. 2016; 22(7): 468–475.	^108^
365155	Saudi Arabia		El Bushra HE, Al Arbash HA, Mohammed M, Abdalla O, Abdallah MN, Al-Mayahi ZK, Assiri AM, BinSaeed AA. Outcome of strict implementation of infection prevention control measures during an outbreak of Middle East respiratory syndrome. Am J Infect Control. 2017; 45(5): 502–507.	^177^
364937	Saudi Arabia		Fagbo SF, Skakni L, Chu DK, Garbati MA, Joseph M, Peiris M, Hakawi AM. Molecular Epidemiology of Hospital Outbreak of Middle East Respiratory Syndrome, Riyadh, Saudi Arabia, 2014. Emerg Infect Dis. 2015; 21(11): 1981-8.	^147^
364620	Mali		Falzarano D, Kamissoko B, de Wit E, Maïga O, Cronin J, Samaké K, Traoré A, Milne-Price S, Munster VJ, Sogoba N, Niang M, Safronetz D, Feldmann H. Dromedary camels in northern Mali have high seropositivity to MERS-CoV. One Health. 2017; 3: 41-3.	^83^
365051	Netherlands		Fanoy EB, van der Sande MA, Kraaij-Dirkzwager M, Dirksen K, Jonges M, van der Hoek W, Koopmans MP, van der Werf D, Sonder G, van der Weijden C, van der Heuvel J, Gelinck L, Bouwhuis JW, van Gageldonk-Lafeber AB. Travel-related MERS-CoV cases: an assessment of exposures and risk factors in a group of Dutch travellers returning from the Kingdom of Saudi Arabia, May 2014. Emerg Themes Epidemiol. 2014; 11: 16.	^161^
364712	Qatar		Farag EA, Reusken CB, Haagmans BL, Mohran KA, Stalin Raj V, Pas SD, Voermans J, Smits SL, Godeke GJ, Al-Hajri MM, Alhajri FH, Al-Romaihi HE, Ghobashy H, El-Maghraby MM, El-Sayed AM, Al Thani MH, Al-Marri S, Koopmans MP. High proportion of MERS-CoV shedding dromedaries at slaughterhouse with a potential epidemiological link to human cases, Qatar 2014. Infect Ecol Epidemiol. 2015; 5: 28305.	^95^
364279	Saudi Arabia		Garbati MA, Fagbo SF, Fang VJ, Skakni L, Joseph M, Wani TA, Cowling BJ, Peiris M, Hakawi A. A Comparative Study of Clinical Presentation and Risk Factors for Adverse Outcome in Patients Hospitalised with Acute Respiratory Disease Due to MERS Coronavirus or Other Causes. PLoS One. 2016; 11(11): e0165978.	^103^
365082	South Korea		Go YY, Kim YS, Cheon S, Nam S, Ku KB, Kim M, Cho NH, Park H, Alison Lee PY, Lin YC, Tsai YL, Thomas Wang HT, Balasuriya UBR. Evaluation and Clinical Validation of Two Field-Deployable Reverse Transcription-Insulated Isothermal PCR Assays for the Detection of the Middle East Respiratory Syndrome-Coronavirus. J Mol Diagn. 2017; 19(6): 817-27.	^208^
364318	China		Guan WD, Mok CK, Chen ZL, Feng LQ, Li ZT, Huang JC, Ke CW, Deng X, Ling Y, Wu SG, Niu XF, Perera RA, Da Xu Y, Zhao J, Zhang LQ, Li YM, Chen RC, Peiris M, Chen L, Zhong NS. Characteristics of Traveler with Middle East Respiratory Syndrome, China, 2015. Emerg Infect Dis. 2015; 21(12): 2278-80.	^41^
364326	France		Guery B, Poissy J, el Mansouf L, Séjourné C, Ettahar N, Lemaire X, Vuotto F, Goffard A, Behillil S, Enouf V, Caro V, Mailles A, Che D, Manuguerra JC, Mathieu D, Fontanet A, van der Werf S, MERS-CoV study group. Clinical features and viral diagnosis of two cases of infection with Middle East Respiratory Syndrome coronavirus: a report of nosocomial transmission. Lancet. 2013; 381(9885): 2265-72.	^3^
364961	Spain		Gutiérrez C, Tejedor-Junco MT, González M, Lattwein E, Renneker S. Presence of antibodies but no evidence for circulation of MERS-CoV in dromedaries on the Canary Islands, 2015. Euro Surveill. 2015; 20(37).	^8^
364917	Qatar		Haagmans BL, Al Dhahiry SH, Reusken CB, Raj VS, Galiano M, Myers R, Godeke GJ, Jonges M, Farag E, Diab A, Ghobashy H, Alhajri F, Al-Thani M, Al-Marri SA, Al Romaihi HE, Al Khal A, Bermingham A, Osterhaus AD, AlHajri MM, Koopmans MP. Middle East respiratory syndrome coronavirus in dromedary camels: an outbreak investigation. Lancet Infect Dis. 2014; 14(2): 140-5.	^7^
365310	Jordan		Habib Z, Asghar F, El Masry K, El Reddy M, Ravi M. MERS-CoV in pregnancy. BJOG. 2015; 122(S1): 274-275.	^232^
394852	Israel		Harcourt JL, Rudoler N, Tamin A, Leshem E, Rasis M, Giladi M, Haynes LM. The prevalence of Middle East respiratory syndrome coronavirus (MERS-CoV) antibodies in dromedary camels in Israel. Zoonoses Public Health. 2018.	^68^
394856	Saudi Arabia		Harrath R, Abu Duhier FM. Sero-prevalence of Middle East respiratory syndrome coronavirus (MERS-CoV) specific antibodies in dromedary camels in Tabuk, Saudi Arabia. J Med Virol. 2018; 90(8): 1285–1289.	^179^
364950	Saudi Arabia		Hastings DL, Tokars JI, Abdel Aziz IZ, Alkhaldi KZ, Bensadek AT, Alraddadi BM, Jokhdar H, Jernigan JA, Garout MA, Tomczyk SM, Oboho IK, Geller AI, Arinaminpathy N, Swerdlow DL, Madani TA. Outbreak of Middle East Respiratory Syndrome at Tertiary Care Hospital, Jeddah, Saudi Arabia, 2014. Emerg Infect Dis. 2016; 22(5): 794–801.	^149^
364837	Saudi Arabia		Hemida MG, Alnaeem A, Chu DK, Perera RA, Chan SM, Almathen F, Yau E, Ng BC, Webby RJ, Poon LL, Peiris M. Longitudinal study of Middle East Respiratory Syndrome coronavirus infection in dromedary camel herds in Saudi Arabia, 2014–2015. Emerg Microbes Infect. 2017; 6(6): e56.	^129^
364835	Saudi Arabia		Hemida MG, Al-Naeem A, Perera RA, Chin AW, Poon LL, Peiris M. Lack of middle East respiratory syndrome coronavirus transmission from infected camels. Emerg Infect Dis. 2015; 21(4): 699–701.	^128^
364868	Saudi Arabia		Hemida MG, Chu DK, Poon LL, Perera RA, Alhammadi MA, Ng HY, Siu LY, Guan Y, Alnaeem A, Peiris M. MERS coronavirus in dromedary camel herd, Saudi Arabia. Emerg Infect Dis. 2014; 20(7): 1231-4.	^135^
394861	Saudi Arabia		Hemida MG, Perera RA, Al Jassim RA, Kayali G, Siu LY, Wang P, Chu KW, Perlman S, Ali MA, Alnaeem A, Guan Y, Poon LL, Saif L, Peiris M. Seroepidemiology of Middle East respiratory syndrome (MERS) coronavirus in Saudi Arabia (1993) and Australia (2014) and characterisation of assay specificity. Euro Surveill. 2014; 19(23).	^180^
364890	Saudi Arabia		Hemida MG, Perera RA, Wang P, Alhammadi MA, Siu LY, Li M, Poon LL, Saif L, Alnaeem A, Peiris M. Middle East Respiratory Syndrome (MERS) coronavirus seroprevalence in domestic livestock in Saudi Arabia, 2010 to 2013. Euro Surveill. 2013; 18(50): 20659.	^138^
365049	United Arab Emirates		Hunter JC, Nguyen D, Aden B, Al Bandar Z, Al Dhaheri W, Abu Elkheir K, Khudair A, Al Mulla M, El Saleh F, Imambaccus H, Al Kaabi N, Sheikh FA, Sasse J, Turner A, Abdel Wareth L, Weber S, Al Ameri A, Abu Amer W, Alami NN, Bunga S, Haynes LM, Hall AJ, Kallen AJ, Kuhar D, Pham H, Pringle K, Tong S, Whitaker BL, Gerber SI, Al Hosani FI. Transmission of Middle East Respiratory Syndrome Coronavirus Infections in Healthcare Settings, Abu Dhabi. Emerg Infect Dis. 2016; 22(4): 647-56.	^228^
394845	Bangladesh		Islam A, Epstein JH, Rostal MK, Islam S, Rahman MZ, Hossain ME, Uzzaman MS, Munster VJ, Peiris M, Flora MS, Rahman M, Daszak P. Middle East Respiratory Syndrome Coronavirus Antibodies in Dromedary Camels, Bangladesh, 2015. Emerg Infect Dis. 2018; 24(5): 926–928.	^38^
364355	South Africa		Ithete NL, Stoffberg S, Corman VM, Cottontail VM, Richards LR, Schoeman MC, Drosten C, Drexler JF, Preiser W. Close relative of human Middle East respiratory syndrome coronavirus in bat, South Africa. Emerg Infect Dis. 2013; 19(10): 1697-9.	^183^
365202	Oman		Jahan F, Al Maqbali AA. The Middle East Respiratory Syndrome Coronavirus (MERS-COV). World Fam Med J. 2015; 13(1): 27-30.	^89^
365126	South Korea		Jeong H, Jeong S, Oh J, Woo SH, So BH, Wee JH, Kim JH, Im JY, Choi SP, Park K, Cho BNH, Hong S. Impact of Middle East respiratory syndrome outbreak on the use of emergency medical resources in febrile patients. Clin Exp Emerg Med. 2017; 4(2): 94–101.	^213^
364342	Egypt		Kandeil A, Shehata MM, El Shesheny R, Gomaa MR, Ali MA, Kayali G. Complete Genome Sequence of Middle East Respiratory Syndrome Coronavirus Isolated from a Dromedary Camel in Egypt. Genome Announc. 2016; 4(2): e00309-16.	^50^
364320	United States		Kapoor M, Pringle K, Kumar A, Dearth S, Liu L, Lovchik J, Perez O, Pontones P, Richards S, Yeadon-Fagbohun J, Breakwell L, Chea N, Cohen NJ, Schneider E, Erdman D, Haynes L, Pallansch M, Tao Y, Tong S, Gerber S, Swerdlow D, Feikin DR. Clinical and laboratory findings of the first imported case of Middle East respiratory syndrome coronavirus to the United States. Clin Infect Dis. 2014; 59(11): 1511-8.	^112^
365064	Saudi Arabia		Kasem S, Qasim I, Al-Doweriej A, Hashim O, Alkarar A, Abu-Obeida A, Saleh M, Al-Hofufi A, Al-Ghadier H, Hussien R, Al-Sahaf A, Bayoumi F, Magouz A. The prevalence of Middle East respiratory Syndrome coronavirus (MERS-CoV) infection in livestock and temporal relation to locations and seasons. J Infect Public Health. 2018.	^166^
365091	Saudi Arabia		Kasem S, Qasim I, Al-Hufofi A, Hashim O, Alkarar A, Abu-Obeida A, Gaafer A, Elfadil A, Zaki A, Al-Romaihi A, Babekr N, El-Harby N, Hussien R, Al-Sahaf A, Al-Doweriej A, Bayoumi F, Poon LLM, Chu DKW, Peiris M, Perera RAPM. Cross-sectional study of MERS-CoV-specific RNA and antibodies in animals that have had contact with MERS patients in Saudi Arabia. J Infect Public Health. 2018; 11(3): 331–338.	^168^
364881	Saudi Arabia		Khalafalla AI, Lu X, Al-Mubarak AI, Dalab AH, Al-Busadah KA, Erdman DD. MERS-CoV in Upper Respiratory Tract and Lungs of Dromedary Camels, Saudi Arabia, 2013–2014. Emerg Infect Dis. 2015; 21(7): 1153-8.	^137^
364288	Saudi Arabia		Khalid I, Alraddadi BM, Dairi Y, Khalid TJ, Kadri M, Alshukairi AN, Qushmaq IA. Acute Management and Long-Term Survival Among Subjects With Severe Middle East Respiratory Syndrome Coronavirus Pneumonia and ARDS. Respir Care. 2016; 61(3): 340-8.	^105^
364933	Saudi Arabia		Khalid M, Khan B, Al Rabiah F, Alismaili R, Saleemi S, Rehan-Khaliq AM, Weheba I, Al Abdely H, Halim M, Nadri QJ, Al Dalaan AM, Zeitouni M, Butt T, Al Mutairy E. Middle Eastern Respiratory Syndrome Corona Virus (MERS CoV): case reports from a tertiary care hospital in Saudi Arabia. Ann Saudi Med. 2014; 34(5): 396–400.	^145^
364271	South Korea		Ki M. 2015 MERS outbreak in Korea: hospital-to-hospital transmission. Epidemiol Health. 2015; 37: e2015033.	^185^
364645	South Korea		Kim HK, Yoon SW, Kim DJ, Koo BS, Noh JY, Kim JH, Choi YG, Na W, Chang KT, Song D, Jeong DG. Detection of Severe Acute Respiratory Syndrome-Like, Middle East Respiratory Syndrome-Like Bat Coronaviruses and Group H Rotavirus in Faeces of Korean Bats. Transbound Emerg Dis. 2016; 63(4): 365-72.	^194^
365180	South Korea		Kim I, Lee JE, Kim K-H, Lee S, Lee K, Mok JH. Successful treatment of suspected organizing pneumonia in a patient with Middle East respiratory syndrome coronavirus infection: a case report. J Thorac Dis. 2016; 8(10): E1190–4.	^202^
365124	South Korea		Kim JE, Heo JH, Kim HO, Song SH, Park SS, Park TH, Ahn JY, Kim MK, Choi JP. Neurological Complications during Treatment of Middle East Respiratory Syndrome. J Clin Neurol. 2017; 13(3): 227–233.	^212^
364628	South Korea		Kim KM, Ki M, Cho SI, Sung M, Hong JK, Cheong HK, Kim JH, Lee SE, Lee C, Lee KJ, Park YS, Kim SW, Choi BY. Epidemiologic features of the first MERS outbreak in Korea: focus on Pyeongtaek St. Mary’s Hospital. Epidemiol Health. 2015; 37: e2015041.	^191^
364642	South Korea		Kim SH, Chang SY, Sung M, Park JH, Bin Kim H, Lee H, Choi JP, Choi WS, Min JY. Extensive Viable Middle East Respiratory Syndrome (MERS) Coronavirus Contamination in Air and Surrounding Environment in MERS Isolation Wards. Clin Infect Dis. 2016; 63(3): 363-9.	^193^
364312	South Korea		Kim SH, Ko JH, Park GE, Cho SY, Ha YE, Kang JM, Kim YJ, Huh HJ, Ki CS, Jeong BH, Park J, Jang JH, Kim WS, Kang CI, Chung DR, Song JH, Peck KR. Atypical presentations of MERS-CoV infection in immunocompromised hosts. J Infect Chemother. 2017; 23(11): 769–773.	^188^
365045	South Korea		Kim T, Jung J, Kim SM, Seo DW, Lee YS, Kim WY, Lim KS, Sung H, Kim MN, Chong YP, Lee SO, Choi SH, Kim YS, Woo JH, Kim SH. Transmission among healthcare worker contacts with a Middle East respiratory syndrome patient in a single Korean centre. Clin Microbiol Infect. 2016; 22(2): e11-e13.	^204^
365093	South Korea		Ko JH, Müller MA, Seok H, Park GE, Lee JY, Cho SY, Ha YE, Baek JY, Kim SH, Kang JM, Kim YJ, Jo IJ, Chung CR, Hahn MJ, Drosten C, Kang CI, Chung DR, Song JH, Kang ES, Peck KR. Serologic responses of 42 MERS-coronavirus-infected patients according to the disease severity. Diagn Microbiol Infect Dis. 2017; 89(2): 106–111.	^209^
365062	South Korea		Ko JH, Seok H, Park GE, Lee JY, Lee JY, Cho SY, Ha YE, Kang JM, Kim YJ, Kang CI, Chung DR, Song JH, Peck KR. Host susceptibility to MERS-CoV infection, a retrospective cohort study of the 2015 Korean MERS outbreak. J Infect Chemother. 2018; 24(2): 150–152.	^205^
364301	South Korea		Korean Society of Infectious Diseases, Korean Society for Healthcare-associated Infection Control and Prevention. An Unexpected Outbreak of Middle East Respiratory Syndrome Coronavirus Infection in the Republic of Korea, 2015. Infect Chemother. 2015; 47(2): 120-2.	^187^
364830	Greece		Kossyvakis A, Tao Y, Lu X, Pogka V, Tsiodras S, Emmanouil M, Mentis AF, Tong S, Erdman DD, Antoniadis A. Laboratory investigation and phylogenetic analysis of an imported Middle East respiratory syndrome coronavirus case in Greece. PLoS One. 2015; 10(4): e0125809.	^61^
364896	Netherlands		Kraaij-Dirkzwager M, Timen A, Dirksen K, Gelinck L, Leyten E, Groeneveld P, Jansen C, Jonges M, Raj S, Thurkow I, van Gageldonk-Lafeber R, van der Eijk A, Koopmans M, MERS-CoV outbreak investigation team of the Netherlands. Middle East respiratory syndrome coronavirus (MERS-CoV) infections in two returning travellers in the Netherlands, May 2014. Euro Surveill. 2014; 19(21).	^84^
364798	Austria		Kwok-ming P, Miu-ling W, Yiu-hong L, Ka-wai S, Liza TM, Shuk-kwan C. International Health Regulations (2005) facilitate communication for in-flight contacts of a Middle East respiratory syndrome case, Hong Kong Special Administrative Region, 2014. West Pac Surveill Response J. 2015; 6(1): 62-5.	^35^
364956	United Arab Emirates		Lau SK, Wernery R, Wong EY, Joseph S, Tsang AK, Patteril NA, Elizabeth SK, Chan KH, Muhammed R, Kinne J, Yuen KY, Wernery U, Woo PC. Polyphyletic origin of MERS coronaviruses and isolation of a novel clade A strain from dromedary camels in the United Arab Emirates. Emerg Microbes Infect. 2016; 5(12): e128.	^225^
365076	South Korea		Lee JY, Kim G, Lim DG, Jee HG, Jang Y, Joh JS, Jeong I, Kim Y, Kim E, Chin BS. A Middle East respiratory syndrome screening clinic for health care personnel during the 2015 Middle East respiratory syndrome outbreak in South Korea: A single-center experience. Am J Infect Control. 2018; 46(4): 436–440.	^207^
365109	South Korea		Lee JY, Kim YJ, Chung EH, Kim DW, Jeong I, Kim Y, Yun MR, Kim SS, Kim G, Joh JS. The clinical and virological features of the first imported case causing MERS-CoV outbreak in South Korea, 2015. BMC Infect Dis. 2017; 17(1): 498.	^210^
365192	Saudi Arabia		Leitmeyer KC. Editorial Commentary: Critical Contribution of Laboratories to Outbreak Response Support for Middle East Respiratory Syndrome Coronavirus. Clin Infect Dis. 2015; 60(3): 378–80.	^34^
364414	Italy		Lelli D, Papetti A, Sabelli C, Rosti E, Moreno A, Boniotti MB. Detection of coronaviruses in bats of various species in Italy. Viruses. 2013; 5(11): 2679-89.	^68^
365097	United Arab Emirates		Li Y, Khalafalla AI, Paden CR, Yusof MF, Eltahir YM, Al Hammadi ZM, Tao Y, Queen K, Hosani FA, Gerber SI, Hall AJ, Al Muhairi S, Tong S. Identification of diverse viruses in upper respiratory samples in dromedary camels from United Arab Emirates. PLoS One. 2017; 12(9): e0184718.	^231^
364877	Kenya		Liljander A, Meyer B, Jores J, Müller MA, Lattwein E, Njeru I, Bett B, Drosten C, Corman VM. MERS-CoV Antibodies in Humans, Africa, 2013–2014. Emerg Infect Dis. 2016; 22(6): 1086-9.	^77^
365188	China		Ling Y, Qu R, Luo Y. [Clinical analysis of the first patient with imported Middle East respiratory syndrome in China]. Chin Crit Care Med. 2015; 27(8): 630–4.	^49^
365099	United States		Lippold SA, Objio T, Vonnahme L, Washburn F, Cohen NJ, Chen TH, Edelson PJ, Gulati R, Hale C, Harcourt J, Haynes L, Jewett A, Jungerman R, Kohl KS, Miao C, Pesik N, Regan JJ, Roland E, Schembri C, Schneider E, Tamin A, Tatti K, Alvarado-Ramy F. Conveyance Contact Investigation for Imported Middle East Respiratory Syndrome Cases, United States, May 2014. Emerg Infect Dis. 2017; 23(9): 1585–1589.	^170^
365074	China		Liu P, Shi L, Zhang W, He J, Liu C, Zhao C, Kong SK, Loo JFC, Gu D, Hu L. Prevalence and genetic diversity analysis of human coronaviruses among cross-border children. Virol J. 2017; 14(1): 230.	^45^
364340	China		Lu R, Wang Y, Wang W, Nie K, Zhao Y, Su J, Deng Y, Zhou W, Li Y, Wang H, Wang W, Ke C, Ma X, Wu G, Tan W. Complete Genome Sequence of Middle East Respiratory Syndrome Coronavirus (MERS-CoV) from the First Imported MERS-CoV Case in China. *Genome Announc*. 2015; 3(4): e00818-15.	^42^
365078	China		Ma X, Liu F, Liu L, Zhang L, Lu M, Abudukadeer A, Wang L, Tian F, Zhen W, Yang P, Hu K. No MERS-CoV but positive influenza viruses in returning Hajj pilgrims, China, 2013–2015. BMC Infect Dis. 2017; 17(1): 715.	^46^
364644	France		Mailles A, Blanckaert K, Chaud P, van der Werf S, Lina B, Caro V, Campese C, Guéry B, Prouvost H, Lemaire X, Paty MC, Haeghebaert S, Antoine D, Ettahar N, Noel H, Behillil S, Hendricx S, Manuguerra JC, Enouf V, La Ruche G, Semaille C, Coignard B, Lévy-Bruhl D, Weber F, Saura C, Che D, investigation team. First cases of Middle East Respiratory Syndrome Coronavirus (MERS-CoV) infections in France, investigations and implications for the prevention of human-to-human transmission, France, May 2013. Euro Surveill. 2013; 18(24).	^58^
364909	United Arab Emirates		Malik A, El Masry KM, Ravi M, Sayed F. Middle East Respiratory Syndrome Coronavirus during Pregnancy, Abu Dhabi, United Arab Emirates, 2013. Emerg Infect Dis. 2016; 22(3): 515-7.	^224^
364902	Saudi Arabia		Memish ZA, Al-Tawfiq JA, Alhakeem RF, Assiri A, Alharby KD, Almahallawi MS, Alkhallawi M. Middle East respiratory syndrome coronavirus (MERS-CoV): A cluster analysis with implications for global management of suspected cases. Travel Med Infect Dis. 2015; 13(4): 311-4.	^139^
364907	Saudi Arabia		Memish ZA, Al-Tawfiq JA, Assiri A, AlRabiah FA, Al Hajjar S, Albarrak A, Flemban H, Alhakeem RF, Makhdoom HQ, Alsubaie S, Al-Rabeeah AA. Middle East respiratory syndrome coronavirus disease in children. Pediatr Infect Dis J. 2014; 33(9): 904-6.	^182^
421188	Saudi Arabia		Memish ZA, Zumla Al, Al-Hakeem RF, Al-Rabeeah AA, Stephens GM. Family cluster of Middle East respiratory syndrome coronavirus infections. N Engl J Med. 2013; 368(26):2487-94.	^110^
364716	Saudi Arabia		Memish ZA, Cotten M, Meyer B, Watson SJ, Alsahafi AJ, Al Rabeeah AA, Corman VM, Sieberg A, Makhdoom HQ, Assiri A, Al Masri M, Aldabbagh S, Bosch BJ, Beer M, Müller MA, Kellam P, Drosten C. Human infection with MERS coronavirus after exposure to infected camels, Saudi Arabia, 2013. Emerg Infect Dis. 2014; 20(6): 1012-5.	^125^
364338	Saudi Arabia		Memish ZA, Cotten M, Watson SJ, Kellam P, Zumla A, Alhakeem RF, Assiri A, Rabeeah AA, Al-Tawfiq JA. Community case clusters of Middle East respiratory syndrome coronavirus in Hafr Al-Batin, Kingdom of Saudi Arabia: a descriptive genomic study. Int J Infect Dis. 2014; 23: 63-8.	^116^
365043	United Arab Emirates		Meyer B, Juhasz J, Barua R, Das Gupta A, Hakimuddin F, Corman VM, Müller MA, Wernery U, Drosten C, Nagy P. Time Course of MERS-CoV Infection and Immunity in Dromedary Camels. Emerg Infect Dis. 2016; 22(12): 2171–2173.	^227^
364303	United Arab Emirates		Meyer B, Müller MA, Corman VM, Reusken CB, Ritz D, Godeke GJ, Lattwein E, Kallies S, Siemens A, van Beek J, Drexler JF, Muth D, Bosch BJ, Wernery U, Koopmans MP, Wernery R, Drosten C. Antibodies against MERS coronavirus in dromedary camels, United Arab Emirates, 2003 and 2013. Emerg Infect Dis. 2014; 20(4): 552-9.	^220^
364970	Burkina Faso, Ethiopia, Morocco		Miguel E, Chevalier V, Ayelet G, Ben Bencheikh MN, Boussini H, Chu DK, El Berbri I, Fassi-Fihri O, Faye B, Fekadu G, Grosbois V, Ng BC, Perera RA, So TY, Traore A, Roger F, Peiris M. Risk factors for MERS coronavirus infection in dromedary camels in Burkina Faso, Ethiopia, and Morocco, 2015. Euro Surveill. 2017; 22(13).	^32^
364958	Saudi Arabia		Mohd HA, Memish ZA, Alfaraj SH, McClish D, Altuwaijri T, Alanazi MS, Aloqiel SA, Alenzi AM, Bafaqeeh F, Mohamed AM, Aldosari K, Ghazal S. Predictors of MERS-CoV infection: A large case control study of patients presenting with ILI at a MERS-CoV referral hospital in Saudi Arabia. Travel Med Infect Dis. 2016; 14(5): 464–470.	^151^
364652	Iran		Moniri A, Marjani M, Tabarsi P, Yadegarynia D, Nadji SA. Health Care Associated Middle East Respiratory Syndrome (MERS): A Case from Iran. Tanaffos. 2015; 14(4): 262-7.	^64^
364796	South Korea		Moon SY, Son JS. Infectivity of an Asymptomatic Patient With Middle East Respiratory Syndrome Coronavirus Infection. Clin Infect Dis. 2017; 64(10): 1457–1458.	^196^
365173	Saudi Arabia		Motabi IH, Zaidi SZA, Ibrahim MH, Tailor IK, Alshehry NF, AlGhamdi MS, Iqbal S, Mudaibigh S, Alnajjar FH. Report of Middle East Respiratory Syndrome Coronavirus (MERS-CoV) Infection in Four Patients with Hematological Malignancies Treated at King Fahad Medical City, Riyadh, Saudi Arabia. Blood. 2016; 128(22): 4903.	^154^
364632	United Arab Emirates		Muhairi SA, Hosani FA, Eltahir YM, Mulla MA, Yusof MF, Serhan WS, Hashem FM, Elsayed EA, Marzoug BA, Abdelazim AS. Epidemiological investigation of Middle East respiratory syndrome coronavirus in dromedary camel farms linked with human infection in Abu Dhabi Emirate, United Arab Emirates. Virus Genes. 2016; 52(6): 848-54.	^222^
364870	Egypt, Somalia, Sudan		Müller MA, Corman VM, Jores J, Meyer B, Younan M, Liljander A, Bosch BJ, Lattwein E, Hilali M, Musa BE, Bornstein S, Drosten C. MERS coronavirus neutralizing antibodies in camels, Eastern Africa, 1983–1997. Emerg Infect Dis. 2014; 20(12): 2093-5.	^52^
364963	Saudi Arabia		Müller MA, Meyer B, Corman VM, Al-Masri M, Turkestani A, Ritz D, Sieberg A, Aldabbagh S, Bosch BJ, Lattwein E, Alhakeem RF, Assiri AM, Albarrak AM, Al-Shangiti AM, Al-Tawfiq JA, Wikramaratna P, Alrabeeah AA, Drosten C, Memish ZA. Presence of Middle East respiratory syndrome coronavirus antibodies in Saudi Arabia: a nationwide, cross-sectional, serological study. Lancet Infect Dis. 2015; 15(5): 559-64.	^152^
364941	Kenya		Munyua P, Corman VM, Bitek A, Osoro E, Meyer B, Müller MA, Lattwein E, Thumbi SM, Murithi R, Widdowson MA, Drosten C, Njenga MK. No Serologic Evidence of Middle East Respiratory Syndrome Coronavirus Infection Among Camel Farmers Exposed to Highly Seropositive Camel Herds: A Household Linked Study, Kenya, 2013. Am J Trop Med Hyg. 2017; 96(6): 1318–1324.	^78^
365066	Bangladesh		Muraduzzaman AKM, Khan MH, Parveen R, Sultana S, Alam AN, Akram A, Rahman M, Shirin T. Event based surveillance of Middle East Respiratory Syndrome Coronavirus (MERS- CoV) in Bangladesh among pilgrims and travelers from the Middle East: An update for the period 2013–2016. PLoS One. 2018; 13(1): e0189914.	^37^
364706	South Korea		Nam HS, Park JW, Ki M, Yeon MY, Kim J, Kim SW. High fatality rates and associated factors in two hospital outbreaks of MERS in Daejeon, the Republic of Korea. Int J Infect Dis. 2017; 58: 37–42.	^195^
365105	Saudi Arabia		Nazer RI. Outbreak of Middle East Respiratory Syndrome-Coronavirus Causes High Fatality After Cardiac Operations. Ann Thorac Surg. 2017; 104(2): e127-e129.	^172^
364330	United Arab Emirates		Ng DL, Al Hosani F, Keating MK, Gerber SI, Jones TL, Metcalfe MG, Tong S, Tao Y, Alami NN, Haynes LM, Mutei MA, Abdel-Wareth L, Uyeki TM, Swerdlow DL, Barakat M, Zaki SR. Clinicopathologic, Immunohistochemical, and Ultrastructural Findings of a Fatal Case of Middle East Respiratory Syndrome Coronavirus Infection in the United Arab Emirates, April 2014. Am J Pathol. 2016; 186(3): 652-8.	^221^
364353	Saudi Arabia		Noorwali AA, Turkistani AM, Asiri SI, Trabulsi FA, Alwafi OM, Alzahrani SH, Rashid MM, Hegazy SA, Alzaydi MD, Bawakid KO. Descriptive epidemiology and characteristics of confirmed cases of Middle East respiratory syndrome coronavirus infection in the Makkah Region of Saudi Arabia, March to June 2014. Ann Saudi Med. 2015; 35(3): 203-9.	^118^
364894	Oman		Nowotny N, Kolodziejek J. Middle East respiratory syndrome coronavirus (MERS-CoV) in dromedary camels, Oman, 2013. Euro Surveill. 2014; 19(16): 20781.	^88^
364255	Saudi Arabia		Oboho IK, Tomczyk SM, Al-Asmari AM, Banjar AA, Al-Mugti H, Aloraini MS, Alkhaldi KZ, Almohammadi EL, Alraddadi BM, Gerber SI, Swerdlow DL, Watson JT, Madani TA. 2014 MERS-CoV outbreak in Jeddah–a link to health care facilities. N Engl J Med. 2015; 372(9): 846-54.	^101^
365122	Saudi Arabia		Olarinmoye AO, Olugasa BO, Niphuis H, Herwijnen RV, Verschoor E, Boug A, Ishola OO, Buitendijk H, Fagrouch Z, Al-Hezaimi K. Serological evidence of coronavirus infections in native hamadryas baboons (Papio hamadryas hamadryas) of the Kingdom of Saudi Arabia. Epidemiol Infect. 2017; 145(10): 2030–2037.	^174^
364282	Saudi Arabia		Omrani AS, Matin MA, Haddad Q, Al-Nakhli D, Memish ZA, Albarrak AM. A family cluster of Middle East Respiratory Syndrome Coronavirus infections related to a likely unrecognized asymptomatic or mild case. Int J Infect Dis. 2013; 17(9): e668-72.	^104^
365072	United Arab Emirates		Paden CR, Yusof MFBM, Al Hammadi ZM, Queen K, Tao Y, Eltahir YM, Elsayed EA, Marzoug BA, Bensalah OKA, Khalafalla AI, Al Mulla M, Khudhair A, Elkheir KA, Issa ZB, Pradeep K, Elsaleh FN, Imambaccus H, Sasse J, Weber S, Shi M, Zhang J, Li Y, Pham H, Kim L, Hall AJ, Gerber SI, Al Hosani FI, Tong S, Al Muhairi SSM. Zoonotic origin and transmission of Middle East respiratory syndrome coronavirus in the UAE. Zoonoses Public Health. 2018; 65(3): 322–333.	^90^
364347	South Korea		Park GE, Ko JH, Peck KR, Lee JY, Lee JY, Cho SY, Ha YE, Kang CI, Kang JM, Kim YJ, Huh HJ, Ki CS, Lee NY, Lee JH, Jo IJ, Jeong BH, Suh GY, Park J, Chung CR, Song JH, Chung DR. Control of an Outbreak of Middle East Respiratory Syndrome in a Tertiary Hospital in Korea. Ann Intern Med. 2016; 165(2): 87–93.	^5^
365131	South Korea		Park JW, Lee KJ, Lee KH, Lee SH, Cho JR, Mo JW, Choi SY, Kwon GY, Shin JY, Hong JY, Kim J, Yeon MY, Oh JS, Nam HS. Hospital Outbreaks of Middle East Respiratory Syndrome, Daejeon, South Korea, 2015. Emerg Infect Dis. 2017; 23(6): 898–905.	^214^
364624	South Korea		Park MH, Kim HR, Choi DH, Sung JH, Kim JH. Emergency cesarean section in an epidemic of the middle east respiratory syndrome: a case report. Korean J Anesthesiol. 2016; 69(3): 287-91.	^190^
364952	South Korea		Park SH, Kim YS, Jung Y, Choi SY, Cho NH, Jeong HW, Heo JY, Yoon JH, Lee J, Cheon S, Sohn KM. Outbreaks of Middle East Respiratory Syndrome in Two Hospitals Initiated by a Single Patient in Daejeon, South Korea. Infect Chemother. 2016; 48(2): 99–107.	^201^
365041	South Korea		Park YS, Lee C, Kim KM, Kim SW, Lee KJ, Ahn J, Ki M. The first case of the 2015 Korean Middle East Respiratory Syndrome outbreak. Epidemiol Health. 2015; 37: e2015049.	^160^
365029	Jordan		Payne DC, Iblan I, Alqasrawi S, Al Nsour M, Rha B, Tohme RA, Abedi GR, Farag NH, Haddadin A, Al Sanhouri T, Jarour N, Swerdlow DL, Jamieson DJ, Pallansch MA, Haynes LM, Gerber SI, Al Abdallat MM, Jordan MERS-CoV Investigation Team. Stillbirth during infection with Middle East respiratory syndrome coronavirus. J Infect Dis. 2014; 209(12): 1870-2.	^75^
365178	Egypt		Perera RA, Wang P, Gomaa MR, El-Shesheny R, Kandeil A, Bagato O, Siu LY, Shehata MM, Kayed AS, Moatasim Y, Li M, Poon LL, Guan Y, Webby RJ, Ali MA, Peiris JS, Kayali G. Seroepidemiology for MERS coronavirus using microneutralisation and pseudoparticle virus neutralisation assays reveal a high prevalence of antibody in dromedary camels in Egypt, June 2013. Euro Surveill. 2013; 18(36): 20574.	^54^
365101	Thailand		Plipat T, Buathong R, Wacharapluesadee S, Siriarayapon P, Pittayawonganon C, Sangsajja C, Kaewpom T, Petcharat S, Ponpinit T, Jumpasri J, Joyjinda Y, Rodpan A, Ghai S, Jittmittraphap A, Khongwichit S, Smith DR, Corman VM, Drosten C, Hemachudha T. Imported case of Middle East respiratory syndrome coronavirus (MERS-CoV) infection from Oman to Thailand, June 2015. Euro Surveill. 2017; 22(33).	^91^
364832	Malaysia		Premila Devi J, Noraini W, Norhayati R, Chee Kheong C, Badrul AS, Zainah S, Fadzilah K, Hirman I, Lokman Hakim S, Noor Hisham A. Laboratory-confirmed case of Middle East respiratory syndrome coronavirus (MERS-CoV) infection in Malaysia: preparedness and response, April 2014. Euro Surveill. 2014; 19(18).	^82^
364825	Italy		Puzelli S, Azzi A, Santini MG, Di Martino A, Facchini M, Castrucci MR, Meola M, Arvia R, Corcioli F, Pierucci F, Baretti S, Bartoloni A, Bartolozzi D, de Martino M, Galli L, Pompa MG, Rezza G, Balocchini E, Donatelli I. Investigation of an imported case of Middle East Respiratory Syndrome Coronavirus (MERS-CoV) infection in Florence, Italy, May to June 2013. Euro Surveill. 2013; 18(34).	^70^
364344	Philippines		Racelis S, de los Reyes VC, Sucaldito MN, Deveraturda I, Roca JB, Tayag E. Contact tracing the first Middle East respiratory syndrome case in the Philippines, February 2015. West Pac Surveill Response J. 2015; 6(3): 3–7.	^93^
364827	Qatar		Raj VS, Farag EA, Reusken CB, Lamers MM, Pas SD, Voermans J, Smits SL, Osterhaus AD, Al-Mawlawi N, Al-Romaihi HE, AlHajri MM, El-Sayed AM, Mohran KA, Ghobashy H, Alhajri F, Al-Thani M, Al-Marri SA, El-Maghraby MM, Koopmans MP, Haagmans BL. Isolation of MERS coronavirus from a dromedary camel, Qatar, 2014. Emerg Infect Dis. 2014; 20(8): 1339-42.	^96^
364900	Jordan		Reusken CB, Ababneh M, Raj VS, Meyer B, Eljarah A, Abutarbush S, Godeke GJ, Bestebroer TM, Zutt I, Muller MA, Bosch BJ, Rottier PJ, Osterhaus AD, Drosten C, Haagmans BL, Koopmans MP. Middle East Respiratory Syndrome coronavirus (MERS-CoV) serology in major livestock species in an affected region in Jordan, June to September 2013. Euro Surveill. 2013; 18(50): 20662.	^74^
364948	Qatar		Reusken CB, Farag EA, Haagmans BL, Mohran KA, Godeke GJ 5th, Raj S, Alhajri F, Al-Marri SA, Al-Romaihi HE, Al-Thani M, Bosch BJ, van der Eijk AA, El-Sayed AM, Ibrahim AK, Al-Molawi N, Müller MA, Pasha SK, Drosten C, AlHajri MM, Koopmans MP. Occupational Exposure to Dromedaries and Risk for MERS-CoV Infection, Qatar, 2013–2014. Emerg Infect Dis. 2015; 21(8): 1422-5.	^99^
364898	Qatar		Reusken CB, Farag EA, Jonges M, Godeke GJ, El-Sayed AM, Pas SD, Raj VS, Mohran KA, Moussa HA, Ghobashy H, Alhajri F, Ibrahim AK, Bosch BJ, Pasha SK, Al-Romaihi HE, Al-Thani M, Al-Marri SA, AlHajri MM, Haagmans BL, Koopmans MP. Middle East respiratory syndrome coronavirus (MERS-CoV) RNA and neutralising antibodies in milk collected according to local customs from dromedary camels, Qatar, April 2014. Euro Surveill. 2014; 19(23).	^98^
364924	Chile, Netherlands, Oman, Spain		Reusken CB, Haagmans BL, Müller MA, Gutierrez C, Godeke GJ, Meyer B, Muth D, Raj VS, Smits-De Vries L, Corman VM, Drexler JF, Smits SL, El Tahir YE, De Sousa R, van Beek J, Nowotny N, van Maanen K, Hidalgo-Hermoso E, Bosch BJ, Rottier P, Osterhaus A, Gortázar-Schmidt C, Drosten C, Koopmans MP. Middle East respiratory syndrome coronavirus neutralising serum antibodies in dromedary camels: a comparative serological study. Lancet Infect Dis. 2013; 13(10): 859-66.	^39^
364651	Nigeria		Reusken CB, Messadi L, Feyisa A, Ularamu H, Godeke GJ, Danmarwa A, Dawo F, Jemli M, Melaku S, Shamaki D, Woma Y, Wungak Y, Gebremedhin EZ, Zutt I, Bosch BJ, Haagmans BL, Koopmans MP. Geographic distribution of MERS coronavirus among dromedary camels, Africa. Emerg Infect Dis. 2014; 20(8): 1370-4.	^57^
364883	Qatar		Reusken CB, Schilp C, Raj VS, De Bruin E, Kohl RH, Farag EA, Haagmans BL, Al-Romaihi H, Le Grange F, Bosch BJ, Koopmans MP. MERS-CoV Infection of Alpaca in a Region Where MERS-CoV is Endemic. Emerg Infect Dis. 2016; 22(6): 1129-31.	^97^
364328	South Korea		Rhee JY, Hong G, Ryu KM. Clinical Implications of 5 Cases of Middle East Respiratory Syndrome Coronavirus Infection in a South Korean Outbreak. Jpn J Infect Dis. 2016; 69(5): 361-6.	^189^
365070	Spain		Rubio E, Martínez MJ, Gonzalo V, Barrachina J, Torner N, Martínez AI, Jané M, Vilella A, Del Rio A, Rodriguez-Valero N, Pinazo MJ, Muñoz J, Soriano A, Trilla A, Vila J, Marcos MÁ. Definitive diagnosis in suspected Middle East Respiratory Syndrome Coronavirus cases. J Travel Med. 2018; 25(1).	^40^
364322	Saudi Arabia		Saad M, Omrani AS, Baig K, Bahloul A, Elzein F, Matin MA, Selim MA, Al Mutairi M, Al Nakhli D, Al Aidaroos AY, Al Sherbeeni N, Al-Khashan HI, Memish ZA, Albarrak AM. Clinical aspects and outcomes of 70 patients with Middle East respiratory syndrome coronavirus infection: a single-center experience in Saudi Arabia. Int J Infect Dis. 2014; 29: 301-6.	^113^
364336	Saudi Arabia		Sabir JS, Lam TT, Ahmed MM, Li L, Shen Y, Abo-Aba SE, Qureshi MI, Abu-Zeid M, Zhang Y, Khiyami MA, Alharbi NS, Hajrah NH, Sabir MJ, Mutwakil MH, Kabli SA, Alsulaimany FA, Obaid AY, Zhou B, Smith DK, Holmes EC, Zhu H, Guan Y. Co-circulation of three camel coronavirus species and recombination of MERS-CoVs in Saudi Arabia. Science. 2016; 351(6268): 81-4.	^115^
365033	Saudi Arabia		Saeed AA, Abedi GR, Alzahrani AG, Salameh I, Abdirizak F, Alhakeem R, Algarni H, El Nil OA, Mohammed M, Assiri AM, Alabdely HM, Watson JT, Gerber SI. Surveillance and Testing for Middle East Respiratory Syndrome Coronavirus, Saudi Arabia, April 2015-February 2016. Emerg Infect Dis. 2017; 23(4): 682–685.	^158^
365023	Pakistan		Saqib M, Sieberg A, Hussain MH, Mansoor MK, Zohaib A, Lattwein E, Müller MA, Drosten C, Corman VM. Serologic Evidence for MERS-CoV Infection in Dromedary Camels, Punjab, Pakistan, 2012–2015. Emerg Infect Dis. 2017; 23(3): 550–551.	^92^
365158	Bahrain		Seddiq N, Al-Qahtani M, Al-Tawfiq JA, Bukamal N. First Confirmed Case of Middle East Respiratory Syndrome Coronavirus Infection in the Kingdom of Bahrain: In a Saudi Gentleman after Cardiac Bypass Surgery. Case Rep Infect Dis. 2017; 2017: 1262838.	^36^
364879	Saudi Arabia		Shalhoub S, Abdraboh S, Palma R, AlSharif H, Assiri N. MERS-CoV in a healthcare worker in Jeddah, Saudi Arabia: an index case investigation. J Hosp Infect. 2016; 93(3): 309-12.	^73^
365031	Saudi Arabia		Shalhoub S, AlZahrani A, Simhairi R, Mushtaq A. Successful recovery of MERS CoV pneumonia in a patient with acquired immunodeficiency syndrome: a case report. J Clin Virol. 2015; 62: 69–71.	^157^
365162	Global		Sharif-Yakan A, Kanj SS. Emergence of MERS-CoV in the Middle East: origins, transmission, treatment, and perspectives. PLOS Pathog. 2014; 10(12): e1004457.	^81^
364911	Saudi Arabia		Sherbini N, Iskandrani A, Kharaba A, Khalid G, Abduljawad M, Al-Jahdali H. Middle East respiratory syndrome coronavirus in Al-Madinah City, Saudi Arabia: Demographic, clinical and survival data. J Epidemiol Glob Health. 2017; 7(1): 29–36.	^140^
365151	Qatar		Sikkema RS, Farag EABA, Himatt S, Ibrahim AK, Al-Romaihi H, Al-Marri SA, Al-Thani M, El-Sayed AM, Al-Hajri M, Haagmans BL, Koopmans MPG, Reusken CBEM. Risk Factors for Primary Middle East Respiratory Syndrome Coronavirus Infection in Camel Workers in Qatar During 2013–2014: A Case-Control Study. J Infect Dis. 2017; 215(11): 1702–1705.	^100^
394858	Nigeria		So RT, Perera RA, Oladipo JO, Chu DK, Kuranga SA, Chan KH, Lau EH, Cheng SM, Poon LL, Webby RJ, Peiris M. Lack of serological evidence of Middle East respiratory syndrome coronavirus infection in virus exposed camel abattoir workers in Nigeria, 2016. Euro Surveill. 2018; 23(32).	^86^
364915	Saudi Arabia		Thabet F, Chehab M, Bafaqih H, Al Mohaimeed S. Middle East respiratory syndrome coronavirus in children. Saudi Med J. 2015; 36(4): 484-6.	^141^
364626	United Kingdom		Thomas HL, Zhao H, Green HK, Boddington NL, Carvalho CF, Osman HK, Sadler C, Zambon M, Bermingham A, Pebody RG. Enhanced MERS coronavirus surveillance of travelers from the Middle East to England. Emerg Infect Dis. 2014; 20(9): 1562-4.	^4^
364273	Greece		Tsiodras S, Baka A, Mentis A, Iliopoulos D, Dedoukou X, Papamavrou G, Karadima S, Emmanouil M, Kossyvakis A, Spanakis N, Pavli A, Maltezou H, Karageorgou A, Spala G, Pitiriga V, Kosmas E, Tsiagklis S, Gkatzias S, Koulouris N, Koutsoukou A, Bakakos P, Markozanhs E, Dionellis G, Pontikis K, Rovina N, Kyriakopoulou M, Efstathiou P, Papadimitriou T, Kremastinou J, Tsakris A, Saroglou G. A case of imported Middle East Respiratory Syndrome coronavirus infection and public health response, Greece, April 2014. Euro Surveill. 2014; 19(16): 20782.	^60^
364709	Jordan		van Doremalen N, Hijazeen ZS, Holloway P, Al Omari B, McDowell C, Adney D, Talafha HA, Guitian J, Steel J, Amarin N, Tibbo M, Abu-Basha E, Al-Majali AM, Munster VJ, Richt JA. High Prevalence of Middle East Respiratory Coronavirus in Young Dromedary Camels in Jordan. Vector Borne Zoonotic Dis. 2017; 17(2): 155-159.	^72^
364622	Qatar		Varughese S, Read JG, Al-Khal A, Abo Salah S, El Deeb Y, Cameron PA. Effectiveness of the Middle East respiratory syndrome-coronavirus protocol in enhancing the function of an Emergency Department in Qatar. Eur J Emerg Med. 2015; 22(5): 316-20.	^94^
364290	United Arab Emirates		Wernery U, Corman VM, Wong EY, Tsang AK, Muth D, Lau SK, Khazanehdari K, Zirkel F, Ali M, Nagy P, Juhasz J, Wernery R, Joseph S, Syriac G, Elizabeth SK, Patteril NA, Woo PC, Drosten C. Acute middle East respiratory syndrome coronavirus infection in livestock Dromedaries, Dubai, 2014. Emerg Infect Dis. 2015; 21(6): 1019-22.	^219^
364284	United Arab Emirates		Wernery U, El Rasoul IH, Wong EY, Joseph M, Chen Y, Jose S, Tsang AK, Patteril NA, Chen H, Elizabeth SK, Yuen KY, Joseph S, Xia N, Wernery R, Lau SK, Woo PC. A phylogenetically distinct Middle East respiratory syndrome coronavirus detected in a dromedary calf from a closed dairy herd in Dubai with rising seroprevalence with age. Emerg Microbes Infect. 2015; 4(12): e74.	^218^
365190	United Arab Emirates		Wernery U. Some epidemiological studies on mers coronavirus in dromedaries in the united arab emirates- a short communication. J Camel Pract Res. 2014; 21(1): 1–4.	^233^
365153	Thailand		Wiboonchutikul S, Manosuthi W, Sangsajja C. Zero Transmission of Middle East Respiratory Syndrome: Lessons Learned From Thailand. Clin Infect Dis. 2017; 64(suppl-2): S167-S170.	^215^
364253	France, Italy, Jordan, Qatar, Saudi Arabia, Tunisia, United Arab Emirates, United Kingdom		Wickramage K, Peiris S, Agampodi SB. “Don’t forget the migrants”: exploring preparedness and response strategies to combat the potential spread of MERS-CoV virus through migrant workers in Sri Lanka. F1000Res. 2013; 2: 163.	^71^
364793	China		Wu J, Yi L, Zou L, Zhong H, Liang L, Song T, Song Y, Su J, Ke C. Imported case of MERS-CoV infection identified in China, May 2015: detection and lesson learned. Euro Surveill. 2015; 20(24).	^43^
365068	South Korea		Xiao S, Li Y, Sung M, Wei J, Yang Z. A study of the probable transmission routes of MERS-CoV during the first hospital outbreak in the Republic of Korea. Indoor Air. 2018; 28(1): 51–63.	^206^
365107	China		Xie Q, Cao Y, Su J, Wu J, Wu X, Wan C, He M, Ke C, Zhang B, Zhao W. Two deletion variants of Middle East respiratory syndrome coronavirus found in a patient with characteristic symptoms. Arch Virol. 2017; 162(8): 2445–2449.	^47^
364928	South Korea		Yang JS, Park S, Kim YJ, Kang HJ, Kim H, Han YW, Lee HS, Kim DW, Kim AR, Heo DR, Kim JA, Kim SJ, Nam JG, Jung HD, Cheong HM, Kim K, Lee JS, Kim SS. Middle East Respiratory Syndrome in 3 Persons, South Korea, 2015. Emerg Infect Dis. 2015; 21(11): 2084-7.	^199^
364888	China		Yang L, Wu Z, Ren X, Yang F, Zhang J, He G, Dong J, Sun L, Zhu Y, Zhang S, Jin Q. MERS-related betacoronavirus in Vespertilio superans bats, China. Emerg Infect Dis. 2014; 20(7): 1260-2.	^44^
364334	Iran		Yavarian J, Rezaei F, Shadab A, Soroush M, Gooya MM, Azad TM. Cluster of Middle East respiratory syndrome coronavirus infections in Iran, 2014. Emerg Infect Dis. 2015; 21(2): 362-4.	^62^
365089	Iran		Yavarian J, Shafiei Jandaghi NZ, Naseri M, Hemmati P, Dadras M, Gouya MM, Mokhtari Azad T. Influenza virus but not MERS coronavirus circulation in Iran, 2013–2016: Comparison between pilgrims and general population. Travel Med Infect Dis. 2018; 21: 51–55.	^65^
364630	Iran		Yousefi M, Dehesh MM, Farokhnia M. Epidemiological and Clinical Characteristics of Patients with Middle East Respiratory Syndrome Coronavirus in Iran in 2014. Jpn J Infect Dis. 2017; 70(1): 115–118.	^63^
364968	United Arab Emirates		Yusof MF, Eltahir YM, Serhan WS, Hashem FM, Elsayed EA, Marzoug BA, Abdelazim AS, Bensalah OK, Al Muhairi SS. Prevalence of Middle East respiratory syndrome coronavirus (MERS-CoV) in dromedary camels in Abu Dhabi Emirate, United Arab Emirates. Virus Genes. 2015; 50(3): 509–13.	^226^
365080	United Arab Emirates		Yusof MF, Queen K, Eltahir YM, Paden CR, Al Hammadi ZMAH, Tao Y, Li Y, Khalafalla AI, Shi M, Zhang J, Mohamed MSAE, Abd Elaal Ahmed MH, Azeez IA, Bensalah OK, Eldahab ZS, Al Hosani FI, Gerber SI, Hall AJ, Tong S, Al Muhairi SS. Diversity of Middle East respiratory syndrome coronaviruses in 109 dromedary camels based on full-genome sequencing, Abu Dhabi, United Arab Emirates. Emerg Microbes Infect. 2017; 6(11): e101.	^230^
364939	Saudi Arabia		Zaki AM, van Boheemen S, Bestebroer TM, Osterhaus AD, Fouchier RA. Isolation of a novel coronavirus from a man with pneumonia in Saudi Arabia. N Engl J Med. 2012; 367(19): 1814-20.	^1^
365103	Saudi Arabia		Zhao J, Alshukairi AN, Baharoon SA, Ahmed WA, Bokhari AA, Nehdi AM, Layqah LA, Alghamdi MG, Al Gethamy MM, Dada AM, Khalid I, Boujelal M, Al Johani SM, Vogel L, Subbarao K, Mangalam A, Wu C, Ten Eyck P, Perlman S, Zhao J. Recovery from the Middle East respiratory syndrome is associated with antibody and T-cell responses. Sci Immunol. 2017; 2(14).	^171^
